# Dissecting Structural and Functional Determinants of Microtubules Stabilization through Guided Chemical Modulation

**DOI:** 10.1021/acs.jmedchem.6c01666

**Published:** 2026-07-30

**Authors:** Francesca Bonato, Rebeca París-Ogáyar, Ahmed Soliman, Óscar Fernández, Beatriz Álvarez-Bernad, Juan Francisco Giménez-Abián, Daniel Lucena-Agell, Rafael Hortigüela, Lisha Singh, Juan Estévez-Gallego, Wei-Shuo Fang, Denisa Ondrúšková, Valle Palomo, Federico Gago, Marcus Braun, Zdeněk Lánský, Shinji Kamimura, J. Fernando Díaz, Roland Brandt, Daniele Passarella, María A. Oliva

**Affiliations:** † Unit for the Development of Biological, Immunological and Chemical Drugs, Centro de Investigaciones Biológicas Margarita Salas − CSIC, Madrid 28040. Spain; ‡ Department of Chemistry, Università degli Studi di Milano, Milan 20133, Italy; § Unit for the Development of Biological, Immunological and Chemical Drugs, IMDEA Nanociencia, Madrid 28049, Spain; ∥ Department of Neurobiology, School of Biology/Chemistry, Osnabrück University, Osnabrück 49076, Germany; ⊥ State Key Laboratory of Bioactive Substances and Functions of Natural Medicines & MHC Key Laboratory of Biosynthesis of Natural Products, Institute of Materia Medica, Chinese Academy of Medical Sciences & Pekin Union Medical College, Beijing 100050, China; # Institute of Biotechnology, Czech Academy of Sciences, BIOCEV, Prague West, Prague 252-50, Czech Republic; ∇ Department of Biomedical Sciences and IQM-UAH Associate Unit, University of Alcalá, Alcalá de Henares 28805, Spain; ○ Department of Biological Sciences, Faculty of Science and Engineering, 83546 Chuo University, Tokyo 192-0393, Japan

## Abstract

Paclitaxel (PTX) is a widely used microtubule (MT) stabilizer whose clinical utility is limited by peripheral neuropathy, likely due to drug-induced structural perturbations of neuronal MTs. Here, we aimed to discern how taxane derivatives modify MT lattice architecture and how structural states regulate motor proteins and MAP dynamics. To decouple MT stabilization from adverse structural changes, we designed, synthesized, and characterized several PTX analogues. Our compound **1b** retains PTX-like stabilizing activity *in vitro* and in cells while preserving a native-like MT lattice. This structural separation allowed direct interrogation of MT structure–function relationship in cells. PTX-induced lattice expansion disrupted dynein-mediated retrograde transport, altered kinesin-1 motility and suppressed dynamic Tau exchange. In contrast, **1b** preserved more physiological Tau dynamics. These findings reveal that MT stabilization and lattice modulation are separable properties and establish drug-imposed MT states as regulators of intracellular transport and MAP behavior.

## Graphical abstract

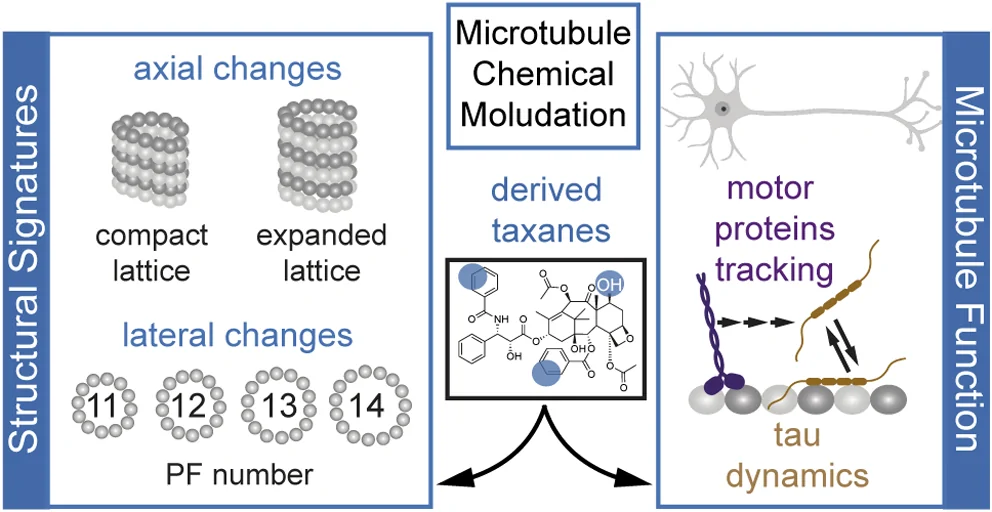

## Introduction

Microtubules (MTs) are dynamic cytoskeletal filaments essential for a wide range of cellular processes in eukaryotic cells. Formed by α/β-tubulin dimers arranged into protofilaments (PFs), they assemble into polarized hollow cylinders that serve as tracks for intracellular transport, organize the mitotic spindle during cell division, and support neuronal development and cell motility. Their characteristic dynamic instability arises from the coordinated regulation of GTP hydrolysis, tubulin isotypes, post-translational modifications, and MT-associated proteins (MAPs), enabling the rapid structural remodeling required for cellular functions.

Because of their central role in mitosis, MTs constitute major targets for anticancer therapy. Drugs that perturb MT dynamics disrupt chromosome segregation and induce apoptosis in rapidly dividing cells. Among them, paclitaxel (PTX), a taxane originally isolated from *Taxus brevifolia*, remains one of the most successful MT-stabilizing agents and is widely used for the treatment of ovarian, breast, and lung cancers, as well as Kaposi’s sarcoma.^1^ PTX suppresses MTs depolymerization by stabilizing the polymer lattice, thereby impairing spindle dynamics during mitosis. However, prolonged PTX treatment frequently causes dose-dependent peripheral neuropathy, highlighting the sensitivity of neuronal MT networks to pharmacological perturbations.^2^ While the molecular basis of this toxicity remains incompletely understood, increasing evidence suggests that MT-stabilizing agents not only suppress MT dynamics but also modify structural and signaling properties of the MT lattice itself.

MTs exhibit intrinsic structural plasticity. *In vitro*, tubulin can assemble into filaments containing between 9 and 16 PFs depending on the geometry of lateral PF interactions.^3^ In cells, the γ-tubulin ring complex typically templates a conserved 13-PFs architecture that optimizes MT straightness and stability.^4^ Nevertheless, specialized tissues can display alternative PF numbers, including activated platelets (14–15 PFs)^5^ and *C. elegans* mechanoreceptor neurons (15-PF MTs),^6^ where post-translational modifications appear to contribute to lattice organization.^7^ MTs also undergo longitudinal conformational transitions associated with lattice expansion and compaction. Expanded lattice states localize preferentially at growing MT ends and influence the recruitment of MAPs.^8^ Though GTP-like expanded lattices correlate with stabilization in the presence of nucleotide analogs, taxanes or tubulin mutants,^9–11^ dynamic MTs can also adopt transiently these conformations.^12^ Notably, PTX and related taxanes can induce persistent lattice expansion throughout the MT shaft,^9,10,13^ raising the possibility that drug-induced structural remodeling alters the interaction of MTs with neuronal MAPs and motors.

This study explores how distinct taxane-induced lattice states influences MT functional properties independently of their ability to stabilize the polymer. Using previously characterized C7-modified taxanes as a structural framework, we designed and synthesized novel PTX derivatives that selectively modulate MT lattice architecture while preserving MT stabilization. This strategy allowed us to experimentally decouple MT stabilization from global lattice expansion. By combining structural analysis, molecular simulations, and a combination of intracellular and *in vitro* transport assays and Tau interaction studies, we show that subtle changes in MT architecture differentially regulate MAP recognition and motor-dependent transport. Together, these findings provide a mechanistic framework for understanding how MT-stabilizing agents reshape MT-dependent cellular processes and establish MT lattice architecture as a functional regulatory element that may guide the future design of MT-stabilizing agents with improved functional selectivity and reduced side effects.

## Results

### Paclitaxel Derivatives at C7 Override Microtubule Lattice Expansion

We first examined whether MT lattice expansion is a general structural response to all taxanes.^12^ To do this, we tested various taxane chemotypes using fluorescent PTX derivatives from our in-house library with modifications at the C2 position (2-debenzoyl-2-(*m*-aminobenzoyl)­paclitaxel (2AP));^14^ C7 (Flutax2 (FTX2) and Hexaflutax (HFX)),^15,16^ and C3′ (3′-N-m-aminobemzamido-3′-N-debenzamidopaclitaxel (3AP),^17^ (Figure 1A). We analyzed the effects of these compounds on the structure of native MTs (assembled in the presence of GTP, thus GDP-bound with GTP caps) using X-ray fiber diffraction of aligned fibers.^10^ This technique allows precise measurement of MTs structural parameters in solution. Briefly, the meridional profile (Figure S1, Figure 1B), provides information on the axial arrangement (i.e., compact vs expanded lattices), while the equatorial profile (Figure 1C) reflects lateral PF associations (i.e., MT diameter, PF number, and distribution of PF variants).

**Figure 1:**
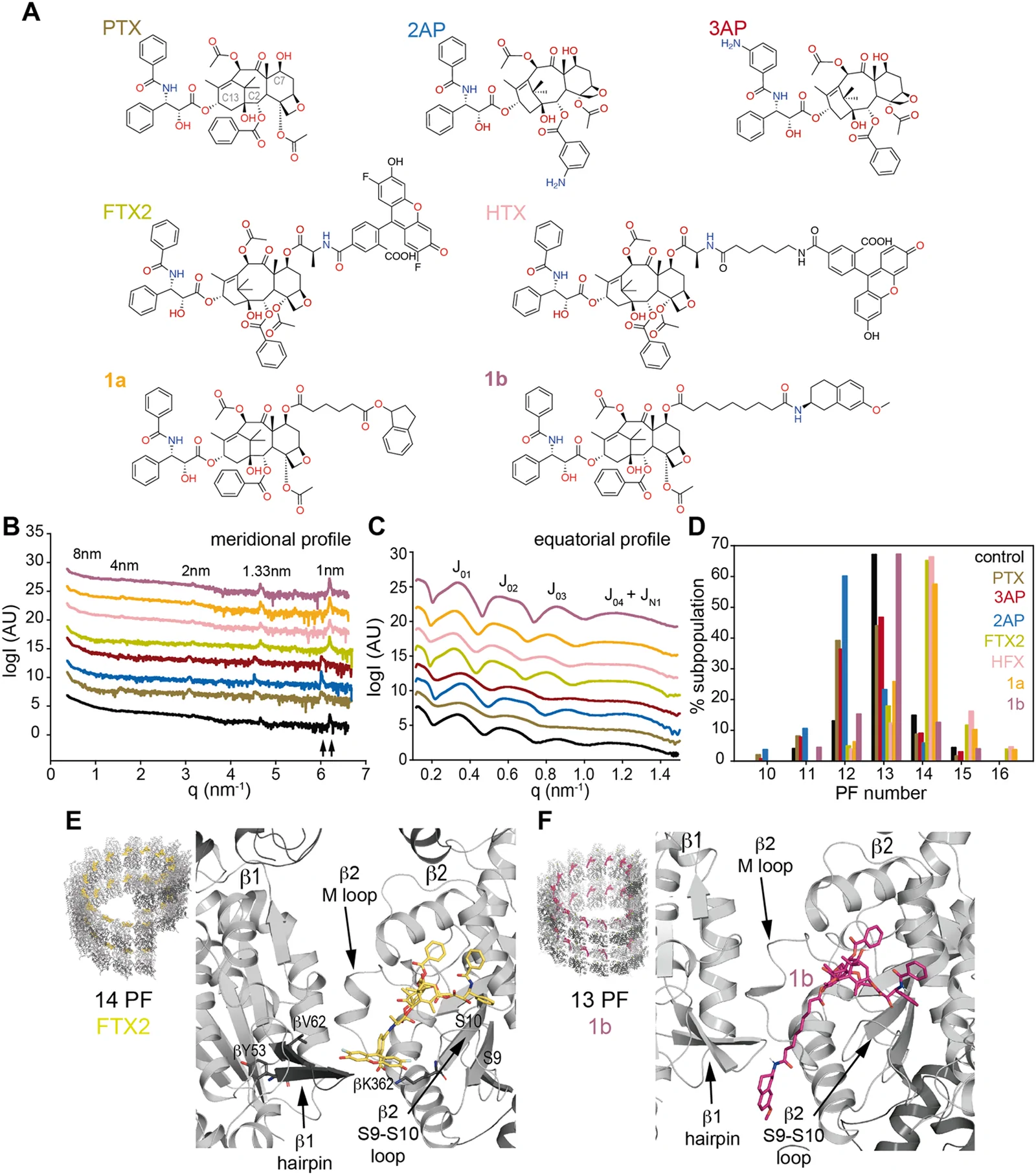
Molecules employed and structural analysis of related stabilized microtubules. (A) Molecules used in this work. (B–C) Fiber diffraction analysis; meridional (B) and equatorial (C) profiles of GTP-assembled MTs (GDP core and GTP cap), and GTP polymerized MTs stabilized with the different compounds. Profiles are shifted in the *y* axis for clarity and Bessel functions are labeled. In B, arrows point to the two possible positions of the 1 nm^–1^ layer band (compact vs expanded lattices). (D) Distribution of MT subpopulations according to the number of PFs in MTs assembled with GTP and MTs further stabilized with the compounds used in this study. (E–F) Left. Fourteen (E)/13 (F) PF MT (PDBs 6DPV and 3JAK, respectively) used in MD simulations and Normal Modes Analysis were built as described in the Methods section. Right. Zoom into the taxane site of one molecule (cartoon representation) showing FTX2 (E)/**1b** (F) (yellow/purple, stick representation) orientation and highlighting lateral interactions. Color code: control (black), PTX (brown), 2AP (blue), 3AP (red) FTX2 (yellow), HFX (salmon), **1a** (orange), **1b** (purple).

Consistent with prior studies, PTX induces axial lattice expansion of GDP-MTs^9,10^ as indicated by a peak position of the third layer line at a *q* = 6.02 (1 nm^–1^, monomer length of 4.17 nm in the real space, Figure 1B and Table 1). Similarly, fluorescence-labeled PTX at C2 (2AP) and C3′ (3AP) induced lattice expansion. Surprisingly, both C7-derived compounds (FTX2 and HFX) promoted a compact lattice conformation (*q* = 6.20 nm^–1^, monomer length of 4.05 nm, Figure 1B), thus overriding this otherwise consistent structural hallmark of taxane binding. Regarding lateral interactions, PTX and its C2 and C3′ derivatives led to the assembly of thinner MTs, whereas C7-modified compounds promoted wider filament formation (Table 1, Figure 1C). In solution, native MTs primarily adopted a 13-PF configuration (>65%). PTX and 3AP induced a mixed population of 12-PF (∼40%) and 13-PF (∼45%) MTs, while 2AP skewed the population toward 12-PF MTs (∼60%). In contrast, C7 derivatives favored the formation of 14-PF MTs (∼65%, Figure 1D).

**Table 1:** Fiber Diffraction Data Analysis^a^

	avg. radius^b^	avg. PF number^c^	1 nm^–1^ band^d^	avg. monomer rise
GDP	11.84 ± 0.02	12.93 ± 0.02	6.19 ± 0.01	4.05 ± 0.01
PTX	11.38 ± 0.03	12.42 ± 0.02	6.01 ± 0.01	4.18 ± 0.01
3AP	11.42 ± 0.03	12.46 ± 0.03	6.01 ± 0.01	4.18 ± 0.01
2AP	11.13 ± 0.02	12.13 ± 0.02	6.01 ± 0.01	4.18 ± 0.01
FTX2	12.57 ± 0.02	13.74 ± 0.02	6.19 ± 0.01	4.06 ± 0.01
HFX	12.77 ± 0.03	13.96 ± 0.03	6.20 ± 0.01	4.05 ± 0.01
**1a**	12.44 ± 0.02	13.57 ± 0.02	6.19 ± 0.01	4.05 ± 0.01
**1b**	11.75 ± 0.01	12.82 ± 0.01	6.19 ± 0.01	4.06 ± 0.01
GMPCPP	12.32 ± 0.01	13.57 ± 0.02	6.02 ± 0.01	4.18 ± 0.01
PTX	11.61 ± 0.05	12.66 ± 0.06	6.02 ± 0.01	4.18 ± 0.01
3AP	11.94 ± 0.02	13.03 ± 0.03	6.02 ± 0.01	4.17 ± 0.01
2AP	12.26 ± 0.02	13.39 ± 0.02	6.02 ± 0.01	4.18 ± 0.01
FTX2	12.96 ± 0.01	14.17 ± 0. 02	6.01 ± 0.01	4.18 ± 0.01
HFX	13.32 ± 0.01	14.57 ± 0.01	6.02 ± 0.01	4.17 ± 0.01
**1a**	13.07 ± 0.02	14.29 ± 0.02	6.02 ± 0.01	4.17 ± 0.01
**1b**	11.93 ± 0.01	13.02 ± 0.01	6.02 ± 0.01	4.17 ± 0.01

a Data are Mean ± SEM.

b nm as calculated from fitting **J**_02_.

c Calculated from interPF mean distance 5.7 nm.^18^

d 2pi nm^–1^ as calculated from 1 nm layer line.

Neither FTX2 nor HFX could override the MT lattice expansion resulting from polymerization in the presence of nonhydrolyzable GTP analog GMPCPP (Table 1, Figures S1 and S2), suggesting that taxane and nucleotide binding sites modulate MT structure via independent mechanisms. Nevertheless, taxane-site stabilization in the presence of GMPCPP increased the average PF number (Table 1, Figure S2) further supporting the proposed crosstalk between the nucleotide and the taxane sites.^12^ Thus, PTX and 3AP moved toward a predominantly 13-PF population (∼65%), while 2AP increased toward a more even distribution of 13-PF (∼40%) and 14-PF (∼40%) MTs. C7 derivatives shifted the population from 14-PF (55% FTX2 and 30% HFX) to 15-PF (30% FTX2 and 50% HFX) MTs (Figure S2).

### New C7-Derivatives to Tailor Microtubule Structural Signaling

PTX structure–activity relationship studies have demonstrated that modifications at C7 are generally well tolerated, as the substitution of the 7-hydroxyl group does not significantly affect activity.^19^ Considering the effect of FTX2 and HFX on lattice compaction while preserving stabilization activity, we selected C7 as a strategically permissive position to dissect how controlled chemical variations at this site modulates MT structure. To investigate this in a systematic manner, we designed two nonfluorescent C7-modified derivatives with defined linker lengths. Our initial hypothesis was that extending the C7 linker could function as a hydrophobic tag capable of interacting with the S9–S10 loop (previously implicated in PTX-induced axial expansion).^12^ At the same time, the terminal substituents were intentionally chosen to be smaller than the bulky fluorophores in FTX2 and HFX to minimize steric perturbations while preserving the ability to modulate local lattice interactions. The resulting compounds incorporate 2,3-dihydro-1H-indene (**1a**) and 6-methoxy-1,2,3,4-tetrahydronaphthalene (**1b**), ring systems (Figure 1A, Figure S3 and Methods).

We first assessed their impact on the cellular microtubular network. Immunofluorescence staining in A549 lung carcinoma cells treated with either compound revealed hallmark features of PTX treatment, including MT bundling, loss of radial MT organization, and perinuclear clearing^20^ (Figure 2A). In addition, cells exhibited aberrant nuclear morphologies, indicative of mitotic failure. These defects manifested as multinucleated cells resulting from multiple abnormal star-like spindles, leading to an aberrant arrangement of chromosomes. Notably, the concentrations required to induce these phenotypes were higher than for PTX (Figure 2A). Cytotoxicity assays in A549 cells (Table 2) revealed that although both compounds retained submicromolar potency, PTX remained more potent. We then evaluated their ability to overcome intrinsic resistance mechanisms using cell lines overexpressing βIII-tubulin isotype (HeLaβIII)^21^ or P-glycoprotein, PgP, (Kb-V1),^21^ compared to parental HeLa and Kb-3.1 cells. Although these new compounds did not fully bypass these resistance mechanisms, they exhibited lower resistance-to-sensitivity (R/S) ratios than did PTX, particularly in βIII-overexpression cells, which is consistent with previous *in vitro* experiments showing that PTX does not expand GDP-MTs assembled from recombinant α1β3 tubulin.^22^ Interestingly, while compound **1b** was more potent in A549 cells, compound **1a** exhibited a lower IC_50_ in HeLaβIII cells, suggesting that the specific βIII-tubulin amino acid substitution pattern may affect **1b** binding more strongly.

**Figure 2:**
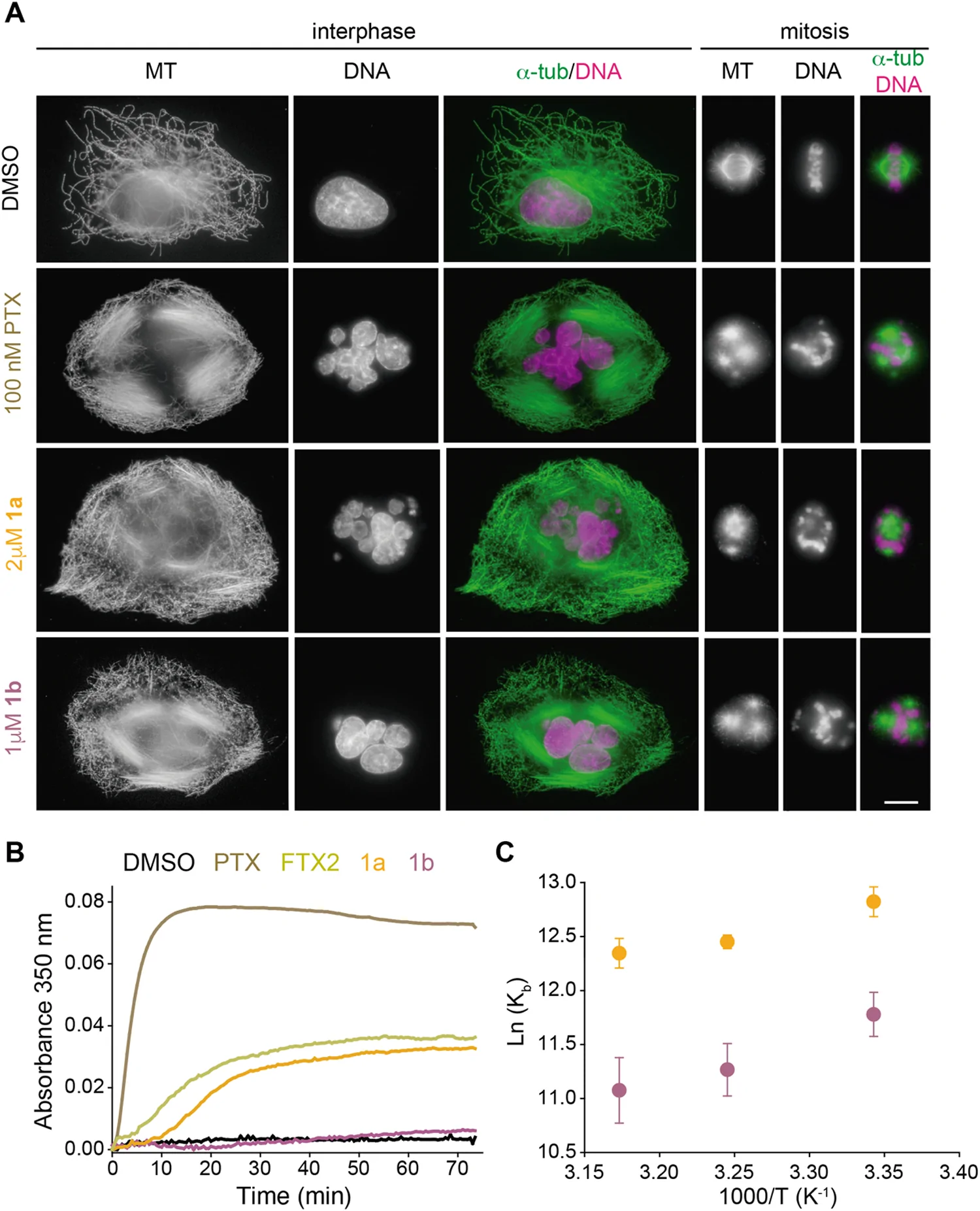
Cellular and biochemical characterization of compounds **1a** and **1b**. (A) Effects of compounds on A549 cells. Each row shows α-tubulin immunostaining (DM1A antibody), DNA labeling (DAPI staining), and the merge of the previous ones (tubulin in green and DNA in magenta) of interphase cells (first three columns) and mitotic cells (following three). Top row panels are control cells treated with 0.5% DMSO (vehicle), with the usual and evenly distributed microtubular network in interphase and normal bipolar spindle with chromosomes aligned at the metaphase plate during mitosis. The following rows display cells treated at selected concentrations showing equivalent effect (PTX 100 nM, **1a** 2 μM and **1b** 1 μM), where the microtubular network is denser and with the presence of bundles during interphase, and a diversity of abnormal mitotic spindle containing misaligned chromosomes. All images are shown at the same magnification; scale bar 10 μm. (B) Time course assembly of 25 μM tubulin in PEDTA buffer in the presence of the vehicle (DMSO, black line) or 35 μM of compound; PTX (brown), FTX2 (yellow), **1a** (orange), **1b** (purple). (C) Van’t Hoff Plot of the equilibrium binding constants of compounds **1a** and **1b** at the assayed temperatures. Error bars represent the standard error of the measurement.

**Table 2:** Comparative Cytotoxicity (IC_50_) of New Synthesized C7-Derivatives^a^

(nM)	A549	HeLa	HeLaβIII	R/S	Kb-3.1	Kb-V1	R/S
PTX	3.1 ± 0.7	1.2 ± 0.4	28.7 ± 0.7	24	2.4 ± 0.2	2700 ± 600	1125
**1a**	510 ± 40	180 ± 20	510 ± 80	3	280 ± 30	>40,000	>142
**1b**	220 ± 20	100 ± 20	720 ± 80	7	150 ± 20	19,000 ± 1000	127

a Data are mean ± SEM.

Next, we evaluated the biochemical properties of these C7-modified compounds. *In vitro* polymerization assays revealed that these new PTX derivatives showed less stabilization potency than does PTX (Figure 2B). Polymerization *v*_max_ were 1.25 × 10^–2^ min^–1^ (PTX), 1.42 × 10^–3^ min^–1^ (FTX2), 1.19 × 10^–3^ min^–1^ (**1a**) and 1.44 × 10^–4^ min^–1^ (**1b**) and hence, the ability of compound **1a** to promote tubulin assembly was similar to that of FTX2, whereas that of **1b** was lower. Preliminary absorption properties predictions using ADMET Lab 3.0 showed poor permeability predictions in the Caco-2 model for both compounds. However, compound **1b** was slightly more permeable, which could be related to a higher cellular uptake for **1b** explaining the differences observed between cellular and *in vitro* assays. Consistently with assembly results, **1a** exhibited ∼10-fold higher binding affinity for the taxane site than did **1b** (Table 3), although the binding constants of both drugs were ∼100-fold lower than that of PTX.^23^

**Table 3:** Equilibrium Binding Constants and Thermodynamic Parameters of New Synthesized C7-Derivatives

	**1a**	**1b**	PTX^b^	FTX2^c^
*K*_b_ (M-1) 26 °C^a^	(3.7 ± 0.2) × 10^5^	(1.3 ± 0.1) × 10^5^	(2.64 ± 0.17) × 10^7^	(5.9 ± 0.3) × 10^7^
*K*_b_ (M-1) 35 °C^a^	(2.6 ± 0.1) × 10^5^	(7.8 ± 0.8) × 10^4^	(1.43 ± 0.17) × 10^7^	(2.2 ± 0.2) × 10^7^
*K*_b_ (M-1) 42 °C^a^	(2.3 ± 0.1) × 10^5^	(6.5 ± 0.9) × 10^4^	(0.94 ± 0.23) × 10^7^	(1.8 ± 0.2) × 10^7^
Δ*G* ^0^ app (kJ mol^–1^) at 26 °C	–31.9 ± 0.1	–28.9 ± 0.3	–41.7 ± 0.2	
Δ*H* ^0^ app (kJ mol^–1^)	–24 ± 4	–35 ± 7	–51.4 ± 4.2	
Δ*S* ^0^ app (J mol^a^K^–1^)	–27 ± 5	20 ± 5	–29.3 ± 13.1	

a Data are the mean ± SEM values of three independent experiments with duplicates in each one.

b As in ref 25.

c As in ref 15. Equilibrium constants from anisotropy measurements.

Analysis of the thermodynamics of binding revealed that the association reactions for both compounds were enthalpy-driven. Taxanes typically do not show any significant changes in heat capacity upon binding,^24^ and Van’t Hoff analysis (Δ*H*_app_^0^) confirmed that binding of these new compounds is exothermic (Figure 2C), consistent with PTX (−51.4 ± 4.2 kJ mol^–1^).^23^ However, compounds **1a** and **1b** display distinct entropic contributions, with **1a** being more entropically favored, possibly due to stronger hydrophobic effects or a lower desolvation penalty upon binding. Overall, these results indicate that (i) both **1a** and **1b** induce MT stabilization, and (ii) either the linker or the bulky moiety modulates the interaction of the PTX core within the taxane site. Moreover, these results reinforce the premise that binding affinity does not directly predict a compound’s ability to promote MT assembly.

To further dissect the contribution of the C7 substituent, we calculated the incremental binding free energy (ΔΔ*G*) associated with PTX-to-derivative modifications at 35 °C. The ΔΔ*G* for **1a** (9.8 kJ mol^–1^) and **1b** (12.8 kJ mol^–1^) were similar to that of HFX (6.9 kJ mol^–1^),^26^ indicating that the C7 modifications introduced are thermodynamically unfavorable. In contrast, FTX2 and to some extent, also Flutax-1, which possesses a Δ*G*_app_^0^ comparable to that of PTX, exhibited neutral to slightly favorable affinity changes (−0.3 and −1.63 kJ mol^–1^).^15^ These findings denote that bulky and negatively charged fluorophores confer more favorable interactions than do smaller organic groups. The discrepancy between HFX and Flutax-1^15^, sharing the same fluorophore, may stem from differences in linker length (alanine aliphatic chain vs single alanine). Together with the differences observed between **1a** and **1b**, these findings suggest that C7 linkers do not behave as productive hydrophobic tags. Instead, shorter flexible substituents yield higher binding affinities for the taxane site, likely because longer hydrophobic chains impose greater desolvation and entropic penalties upon binding.

Finally, we examined the structural effects of **1a** and **1b** on MT using X-ray fiber diffraction. Both compounds, consistently with other C7-derivatives, promoted a compact lattice structure (Figure 1B, Table 1). However, their effects on lateral PFs interactions differed. Compound **1a**, like FTX2 and HFX, promoted wider filaments with a dominant 14-PFs population (∼ 60%, Figure 1D). Remarkably, **1b** did not significantly alter the PF distribution relative to native MTs (>65% 13 PF). Nonetheless, neither compound was able to override the lattice expanding effect of GMPCPP (Figure S2). Interestingly and differently from native conditions, compound **1b** did not show any alteration on the PF distribution in the presence of GMPCPP. This finding denotes that the interaction of this compound disables the crosstalk switch between the nucleotide and the taxane sites.

### Implication of C7 Derivatives Chains on Protofilaments Lateral Interactions

To understand how lattice expansion is overridden by PTX C7 derivatives we used computational methods. Initial molecular dynamics (MD) simulations of ligands FTX2 and **1b** in explicit water under comparable conditions revealed quite distinct conformational preferences (Figure S4A) and a prevalence of poses incompatible with occupancy of the taxane site, in consonance with earlier work on other PTX-related taxanes.^27^ Of note, FTX2 is shown to be able to form an intramolecular hydrogen bond involving the side-chain amide group; this interaction is not feasible in **1b** because the amide group has been separated further from the ester moiety attached to the taxane core by means of a polymethylene linker. This longer hydrophobic chain endows **1b** with much greater conformational freedom, which is translated into a more random orientation of the attached heteroaromatic ring relative to FTX2 (Figure S4A) and correlates with its lower binding affinity.

We next analyzed the dynamic behavior of a fully solvated molecular model of a short PF consisting of three longitudinally αβ-tubulin dimers capped by an α-tubulin molecule,^12^ in both apo- and FTX2-bound forms (Figure S4B). We found that, in the bound conformation, FTX2 maintains the intramolecular hydrogen bond within the β-tubulin taxane pocket reported above and places its fluorophore facing the intradimer α-tubulin subunit below, sitting between the S9–S10 loop and the M loop (Figure S4B). Consistent with our fiber diffraction data, simulations reliably reproduced a compact axial lattice within the dimer (α2:β2) and at the interdimer interface (β2:α3, Figure S4C). The origin of lattice expansion has been attributed to the displacement of the S9–S10 loop, caused by backbone rearrangement at βR369-βG370, which propagates to the attached α-tubulin subunit.^12^ Our MD trajectories show a shift in the O2′ taxane position compared to PTX and other taxanes structures. This change enables O2′ to act as a hydrogen bond acceptor from the amine backbone of βR369, rather than as a donor to the carbonyl backbone. Additionally, the oxetane group (O5) of the taxane hydrogen bonds to the backbone NH of βT276 (M loop), while the amide carbonyl (O4′) accepts a hydrogen bond from the (NE2)­βH229 (central helix H7), in good agreement with previous crystal structures and MD trajectories.^12^

To understand the effect of ligand binding on lateral interactions between PF, we further studied MT patches (Figure S4D). Binding of FTX2 to laterally assembled PFs invariably showed a strong ionic interaction between the free carboxylate of the benzoyl group attached to the fluorophore and the protonated amino group of βK362 (Figure 1E). Thus fixed, the fluorinated xanthene moiety is positioned very close to the twisted β-hairpin (^53^YNEAAGNKYV^62^) of the β subunit in the vicinal PF. This means that FTX2 binding to MTs has an effect on lateral interactions that is more profound than that of PTX and analogues not bearing a long substituent at C7. In the case of **1b**, however, the longer and more flexible linker is capable of rotating quite freely both at the intradimer α1:β1 interface and at the inter-PF space (Figure 1F, Figure S4E).

Normal mode analyses of different 3-PF MT pieces herein described as [(α1:β1-α2:β2)/(α1′:β1′-α2′:β2′)/(α1″:β1″-α2″:β2″)] consistently revealed that the observed ligand-induced changes in PF number can be effectively approximated by the first or second nontrivial normal mode (Movie S1). Thus, depending on the direction (opening vs closing motion), 13-PF MTs can evolve toward narrower 12-PF ones or toward wider MTs consisting of 14–15 PFs in a similar fashion as we showed before for laulimalide- and peloruside-bound MTs.^3^ We posit that this low-frequency motion can have a much larger amplitude than the spontaneous equilibrium thermal fluctuation when a ligand is bound because it is an activated process, *i.e*., ligand binding brings in additional energy that can stretch the structure along the normal mode much further and lock it in this state until ligand dissociation takes place.

### Different Microtubule Structural Signals Drive Anterograde and Retrograde Intracellular Trafficking

Building on our ability to chemically modulate MT structure, we next explored how these changes influence signaling patterns involved in MAPs interactions. Given that MTs are key players in intracellular transport – guiding organelles, vesicles and chromosomes via molecular motors like dyneins and kinesins – we assessed the impact of our compounds on transport dynamics in neuroblastoma SH-SY5Y cells. We combined live-cell imaging with previously developed tracking peptides (Figure 3A): Cy5-DBP, which encompasses the sequence that specifically recognizes Dynein Light Chain LC8 (retrograde transport), and Cy5-KBP, with a sequence that binds to the tetratricopeptide repeat of the kinesin-1 light chain KLC1 (anterograde transport).^28^ Because the aim was to determine the functional consequences of defined drug-imposed MT states rather than intrinsic drug potencies, we applied high concentrations of the compounds (1 μM PTX or 2.5 μM FTX2, **1a** or **1b**) to generate robust MT stabilization phenotypes and define lattice states. However, this was for a short incubation period (1 to 2.5 h, including imaging time) to avoid mitotic arrest or compromise cell viability. We then assessed particle behavior by measuring mean track displacement and mean track velocity.

**Figure 3:**
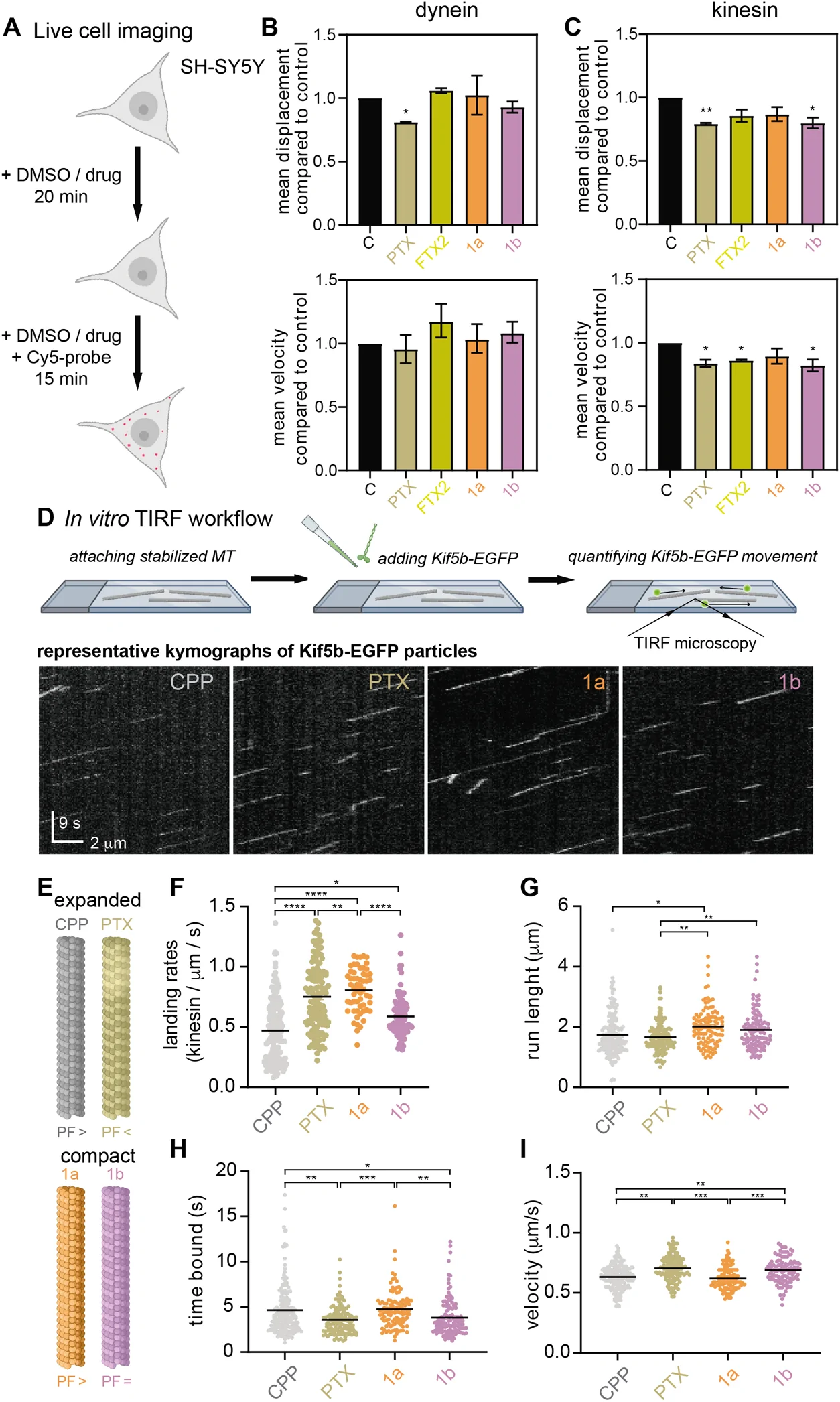
Characterization of motor proteins tracking. (A) Schematic representation of live cell imaging of kinesin and dynein binding probes movement in SH-SY5Y cells. (B–C) Particle movement parameters measured with Cy5-DBP (B) and Cy5-KBP (C) probes in the presence of 1 μM PTX or 2.5 μM FTX2, **1a** or **1b**, in nondifferentiated SH-SY5Y cells. Data are the mean ± SEM values of two independent experiments with two to three different fields analyzed per experiment and statistical significance is as follows (**p < 0.01 and *p < 0.05). (D) Schematic representation of the single-molecule kinesin-1 motility assay. Stabilized MTs were immobilized in the channel, Kif5b-EGFP was added and the motility of single motors was recorded using TIRF microscopy. Representative kymographs display the movement of multiple individual Kif5B-EGFP motors along a single MT; each line corresponds to the trajectory of a single motor. Horizontal scale bar 2 μm; vertical scale bar 9 s. (E) Simplified scheme of how the compounds studied affect MT structure. (F–I) Scatter plots and summary quantification of at least three independent experiments showing landing rate (kinesin/μm/s, (F)), run length (μm, (G)), time bound (s, H) and velocity (μm/s, I) of single Kif5b-EGFP molecules on MTs stabilized by different mechanisms. Each data point represents one motor molecule. Data pooled from *n* ≥ 2 experiments per condition across 12 MTs. Color code: PTX (brown), FTX2 (yellow), **1a** (orange), **1b** (purple), GMPCPP (gray).

Regarding the transport observed with Cy5-DBP (Movies S2–S6), we found that only PTX had a significant effect, reducing particle displacement by approximately 20% compared to nontreated control cells, without affecting speed (Figure 3B, Table S1). These data suggest that dynein can sense lattice expansion, resulting in reduced contact time without compromising motor processivity. In contrast, we found no specific correlation between movement of Cy5-KBP (Movies S7–S11) and either axial or lateral MT lattice modifications (Figure 3C, Table S1). Surprisingly, **1b** (which produces lattices close to the native state, Figure 1D) led to reductions in kinesin binding probe tracking displacement (∼20%) and velocity (∼16%, Table S1) similar to those observed with PTX (which causes lattice expansion and diameter reduction, Figure 1D). Meanwhile, FTX2 (compact lattice with increased diameter) only reduced particle velocity slightly (∼14%). However, **1a** (which induces similar lattice modifications to FTX2, Figure 1D) did not affect particle tracking, which might be related to the lower cellular uptake observed (Figure 2, Tables 2).

To gain mechanistic insight into the kinesin phenotype observed in cells, we reduced system complexity using an *in vitro* reconstitution assay. Specifically, we performed time-lapse imaging using total internal reflection fluorescence (TIRF) microscopy with immobilized and stabilized MTs in a flow chamber, into which we introduced 1.08 nM kinesin-1 motors (constitutively active human Kif5B 1–905-EGFP).^29^ We then analyzed individual kinesin movements to quantify landing rates (number of motors per unit MT length per unit time), run lengths, interaction times, and average velocities (Figure 3D, Movies S12–S15). In contrast to our experiments in cells, here we evaluated each structural modification individually to dissect the effects of MT lattice parameters on kinesin-1 mediated transport. Since nonstabilized MTs rapidly disassemble during the assay, we considered **1b**-stabilized MTs as the most suitable control because they exhibit a lattice structure close to the native form (Table 1). Each compound studied introduced distinct lattice alterations: **1a**-MTs increase the number of PFs in a compact lattice, and GMPCPP-MTs and PTX-MTs combine both axial expansion and an increase (GMPCPP) or decrease (PTX) in diameter (Figure 3E).

In this simplified system, we observed that different kinesin motility patterns responded uniquely to the various lattice changes. Axial expansion (GMPCPP and PTX) did not correlate with time bound, velocity or landing rates (Figure 3H,I,F). Larger MT diameter (GMPCPP and **1a**) increased the time bound (Figure 3H) and decreased the velocity (Figure 3I), while the landing rate did not correlate but decreased on GMPCPP compared with PTX (both expanded) while it increased on **1a** compared with **1b** (both compacted) (Figure 3F). Lateral changes to the MT lattice did not significantly affect run length (Figure 3G) as going slower (Figure 3I) and staying longer (Figure 3H) cancel each other out, and because the impact of axial expansion alone was minor, we conclude that individual lattice alterations are not primary determinants of kinesin motility. However, combined changes in axial spacing and PF number (e.g., **1b** vs PTX; **1a** vs PTX) lead to a more pronounced reduction in run length (Figure 3G).

Contrary to previous reports, where the effect of lattice expansion was assessed by comparing GMPCPP-stabilized MTs with nonstabilized GDP-MTs,^30^ our results show that lattice expansion reduces kinesin-1 affinity for the MT lattice. This discrepancy may arise from the known structural heterogeneity of GDP-MTs (body and cap regions), whereas our system uses structurally homogeneous, stabilized lattices that enable us to isolate and evaluate the effect of discrete and combined structural features on kinesin-1 behavior. Our findings, are also at odds with assays demonstrating that gliding velocities of recombinant-single-tubulin-isotype MTs on kinesin-1 covered surfaces increase on expanded lattices^22^ and that kinesin-1 induces lattice expansion upon movement.^31^ The discrepancy might result either from (a) MTs assembled from mixtures of tubulin isotypes (as used in this work) responding differently to taxanes and GMPCPP than single-tubulin-isotype MTs; (b) synchronized teams of kinesin molecules behaving differently from single kinesins; or (c) from a combination of both. Notably, our findings further support the view that the expanded lattice states induced by GMPCPP and PTX – commonly used as MT-stabilizing agents *in vitro* – are structurally distinct.^9,13,32^ This distinction should be carefully considered in future studies investigating MAPs-MT interactions.

### Both Axial and Lateral Structural Signals Drive Tau Binding and Kinetics

Building on the role of Tau dynamics in axonal MT function,^33,34^ we next used Tau as a structural sensitive neuronal MAP to assess whether drug-imposed MT lattice states alter MAP recognition. To this end, we initially performed fluorescence decay after photoactivation (FDAP) experiments to assess potential changes in the interaction of the longest human Central Nervous System Tau isoform (Tau441) with MTs in axon-like processes of model neurons. We tagged Tau at the N-terminus with photoactivatable GFP (PAGFP) and expressed it exogenously in PC12 cells differentiated into a neuronal phenotype (Figure 4A). After locally activating a subset of labeled Tau molecules in the middle of a process, we monitored FDAP over time in the activated region (Figure 4B). Tau gradually dissipated from the activation site. Notably, only PTX treatment slowed FDAP decay relative to control cells, indicating enhanced Tau binding to MTs in axon-like processes.

**Figure 4:**
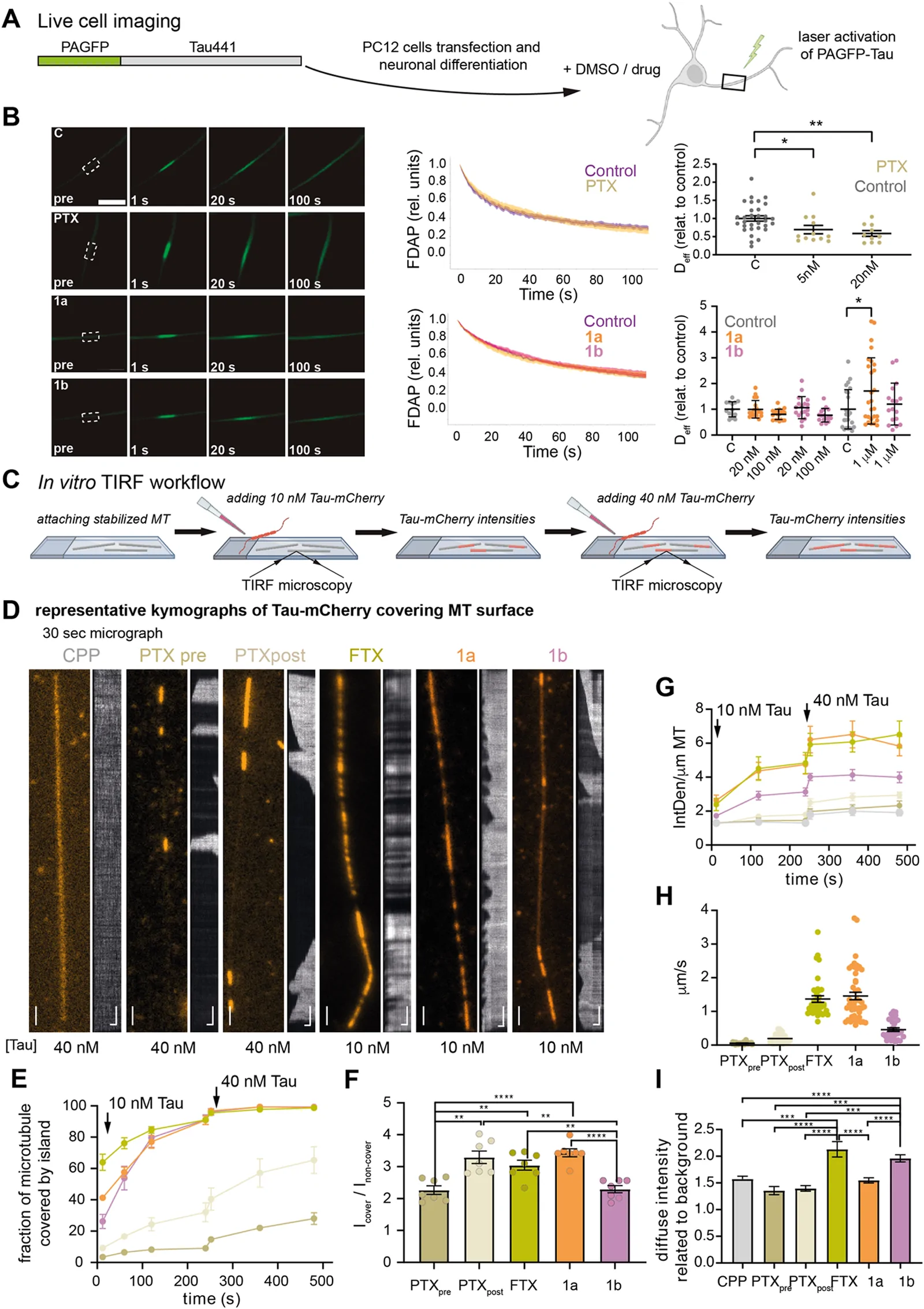
Characterization of Tau interaction with stabilized MTs. (A) Live cell imaging of tau dynamics in axon-like processes of neuronally differentiated PC12 cells. Schematic representations of the expressed Tau construct with the N-terminal PAGFP fusion in green. (B) Left: Representative time-lapse micrographs of a fluorescence decay after photoactivation (FDAP) experiment. Scale bar, 10 μm. The activation and recording region are indicated in the preactivation image (6 μm length). Middle: Curve representing the mean of all individually fitted FDAP curves after photoactivation of PAGFP-Tau-expressing PC12 cells. Mean ± SEM of Tau cells in the presence of PTX, compound **1a** and **1b** are shown (*n* > 100 curves for each condition). Right: Scatterplots of effective diffusion constants (*D*_eff_). Statistically significant differences between treatments as determined by one-way ANOVA followed by a Dunnett’s posthoc test, are indicated. **p < 0.01 and *p < 0.05. (C) Schematic representation of TIRF microscopy assay setup. Label-free stabilized MTs are attached to a glass coverslip. Ten nM purified Tau-mCherry is added and its MT binding recorded in TIRF microscopy during 4 min. 40 nM Tau-mCherry is added and its MT binding recorded in TIRF microscopy for another 4 min. The fluorescence intensity of Tau-mCherry on each MT in the chamber is measured and analyzed. (D) Representative images (at frame 30 s) and their corresponding kymographs of Tau-mCherry binding to MT stabilized with GMPCPP and PTX (40 nM Tau-mCherry) and, FTX2, **1a** and **1b** (10 nM Tau-mCherry). Vertical scale bars 2 μm and horizontal scale bars 60 s. (E) Fraction of MT length covered by Tau envelopes after the addition of Tau-mCherry (F). Mean ratio of intensity of covered regions divided by intensity of noncovered areas upon addition of Tau-mCherry on MTs stabilized with different drugs. (G) Integrated density per MT μm after the addition of Tau-mCherry. (H) velocity (μm/s) of envelope grow upon addition of 10 nM Tau-mCherry on MTs stabilized with different drugs. (I) diffuse intensity signal related to background (low-intensity, nonclustered signal distinct from high-intense envelope structures) measured on MTs stabilized with different drugs. Error bars represent Standard Error of the Mean (SEM), *n* ≥ 2 experiments per condition, 12 MTs. Color code: PTX_pre_ (brown), PTX_post_ (light brown), FTX2 (yellow), **1a** (orange), **1b** (purple), GMPCPP (gray).

To quantify changes in Tau’s effective diffusion constant (*D*_eff_) as a result of its dynamic interactions with MTs, we measured FDAP at various compound concentrations and fitted the resulting decay curves using a previously established reaction-diffusion model.^35^ PTX treatment significantly reduced Tau’s *D*_eff_, with a notable effect already at 5 nM (Figure 4B). This reduction corresponded to a ∼40% decrease in the unbound Tau population (4.4% versus 7.5% at 20 nM PTX), indicating increased MT binding. In contrast, compound **1a** produced the opposite effect, increasing Tau’s *D*_eff_ and therefore reducing MT binding, although only at a very high concentration. Compound **1b**, in turn, did not significantly alter Tau-MT interactions, even at concentrations up to 1 μM, (Figure 4B). These findings suggest that an expanded lattice decreases Tau release dynamics in axon-like processes, whereas compacted and thicker MTs induce by compound **1a** promote increased Tau mobility. Remarkably, the GDP-like MTs stabilized by compound **1b** remain largely neutral with respect to Tau-MT interactions.

Previous studies *in vitro* have shown that Tau molecules strongly prefer compacted GDP lattices.^36^ Tau binds cooperatively to MT surfaces, resulting in a cohesive layer depending on tau-tau interactions, termed MT envelope, which selectively regulates access to MT lattices, and shields MTs from severing enzymes.^36–38^ On expanded PTX-MTs, Tau envelope also forms and the lattice can get locally compacted.^36^ To further explore how MT lattice structure influences Tau binding, we employed an *in vitro* reconstitution assay. We deposited stabilized MTs in a flow chamber, and visualized them via interference reflection microscopy (Figure 4C). We then added 10 nM Tau-mCherry (labeled purified Tau 441 isoform at the C-terminus with mCherry) and monitored its association with MTs using time-lapse TIRF microscopy. After 4 min, we increased Tau concentration to 40 nM, and continued imaging for an additional 4 min. We measured fluorescence intensities along MTs to quantify Tau binding (Movies S16–S27). Consistent with previous studies, Tau bound diffusively along GMPCPP-MTs^38^ and formed high-density envelopes that progressively expanded only on PTX-MTs^37,38^ (Figure 4D). Even at the highest Tau concentration and observation time, we did not notice full MT coverage under these two conditions. Remarkably, we found differences between MTs assembled in the presence of PTX (PTX_pre_) and those stabilized with PTX after polymerization (PTX_post_). PTX_post_-MTs showed greater envelope coverage (up to 60% at 40 nM Tau-mCherry compare to 30% on PTX_pre_-MTs, Figure 4E). PTX_post_-MTs also supported slightly faster envelope growth (0.20 ± 0.02 μm/s versus 0.054 ± 0.006 μm/s for PTX_pre_-MTs, Figure 4H). Further, fiber diffraction experiments under the same experimental conditions used in the TIRF studies (i.e., MTs preparation 48 h beforehand) showed subtle structural differences among these stabilized MTs at the lateral interphase (Table S2). These findings suggest that MT lattice remodeling occurs slowly, at least in lateral PF arrangement, which likely contributes to the Tau binding patterns found.

We detected markedly different Tau binding on compact lattices (i.e., FTX2-, **1a-**, and **1b**-MTs). At 40 nM, Tau-mCherry fully coated these stabilized MTs, further supporting that Tau interaction is favored on compact lattices as previously described.^36^ When we reduced the concentration to 10 nM, we could distinguish the two kinetic distinct phases of Tau binding reported earlier:^37^ low-density regions with rapid turnover and high-density envelopes with slow turnover (Figure 4D–H). At this lower concentration, PTX-MTs primarily displayed low-density Tau binding, with minimal high-density envelopes formation, especially on PTX_pre_-MTs (Figure 4E,G). Therefore, to better capture Tau kinetics across all conditions, we used a two-step protocol: we first added 10 nM Tau-mCherry, followed by 40 nM. Under these conditions, Tau-mCherry bound similarly to FTX2- and **1a**-MTs, suggesting that Tau recognizes these alike lattices in the same way (Figure 4D–G). Interestingly, **1b**-MTs together with FTX2 showed higher diffuse fluorescence intensity than other stabilized MTs (Figure 4I), likely indicating a more favorable noncooperative and rapid turnover Tau binding. Otherwise, wider MTs promoted cooperative envelope formation, with an initial envelope coverage of 60% (FTX2-MTs) and 40% (**1a**-MTs) versus only 20% (**1b**-MTs, Figure 4E)), and envelope growth rates of 1.37 ± 0.09 μm/s in FTX2-MTs and 1.5 ± 0.1 μm/s in **1a**-MTs compared to 0.46 ± 0.05 μm/s in **1b**-MTs (Figure 4H). Since the cohesiveness of these envelopes depends on cooperative interactions through Tau N-^37^ and C-terminus,^38^ the wider angle between PFs and also the different PF skews in FTX2- and **1a**-MTs might be favoring these interactions, though the specific mechanism remains elusive.

## Discussion and Conclusions

PTX is one of the most relevant drugs in cancer therapy, but its use is hampered by the appearance of peripheral neuropathy, the most common dose-limiting side effect observed in patients. Although the precise mechanism underlying this pathology remains unclear, it is likely related to PTX’s interference with neuronal MTs rather than from off-target toxicity.^2^ Neuronal MTs are intrinsically more stable due to the expression of less dynamic tubulin isotypes and the presence of specialized MAPs. Thus, PTX-induced stabilization may affect MT function differently in neurons. The main characteristic effects of the molecular mechanism of action of PTX are the structural distortion induced by axial lattice expansion and a reduction in PF number.^9,10^ Importantly, not all clinically used taxanes impose identical structural effects on MTs. Docetaxel (DTX), another approved taxane, despite sharing with PTX a very similar structure, the taxane-binding site and induced longitudinal lattice expansion, differs substantially from the biochemical and pharmacological points of view. DTX is a more efficient promoter of tubulin assembly, a more potent MT stabilizer and clinically exhibits lower frequency and severity of peripheral neuropathy. The main difference between these two taxanes is observed on the differential effect on lateral interfaces, which is minimal in DTX-bound MTs whereas significantly reduces MT diameter in PTX-bound MTs.^39,12,40^ Therefore, neurotoxicity may not arise merely from MT stabilization, but rather from the specific combination of axial and lateral lattice states imposed by each compound. It is known that deviations from the canonical 13-PF geometry can induce PF supertwisting around the MTs,^41^ and the conformational state of tubulin within MTs is known to modulate the interaction of MAPs such as VASH1/SVBP^42^ or doublecortin.^43^ Given that the MT structure serves as a key signaling platform for cellular recruitment of motor proteins and MAPs, it becomes evident that such structural distortions, which in addition spread along the fiber, may underlie MT malfunction by altering their recognition patterns in axons following PTX exposure.

To test this hypothesis, we set out to design a compound capable of exerting PTX-like activity without inducing structural distortion of MTs, and to investigate the cellular effects of such compounds. Based on our initial X-ray fiber diffraction results – and considering that modifications at the C7 position of PTX have shown potential to modulate its biological activity^44^ – we designed, synthetized and characterized novel compounds that allowed us to specifically tailor MT structure. These compounds retained the ability to stabilize MTs both *in vitro* and in cells, although with reduced potency, which we then correlated with differences in linker length and attached moieties. Notably, compound **1b** emerged as the only derivative able to mimic a native-like MT structure while maintaining MT stabilization activity. This finding prompted us to explore which structural features of MT drive MAP recognition. Our *in vitro* characterization provided the best up-to-date mechanistic insight, while cellular studies integrated this knowledge into a more complex and heterogeneous context, shaped by drug-specific parameters (resulting in nonequimolar intracellular concentrations), the “tubulin code” – such as variations in tubulin isotypes that also regulate the PF number^45^ – and the presence of additional MAPs. Because stabilized MTs exhibit quasi-homogeneous structures, our approach enabled the identification of how individual structural cues affect intracellular transport and modulate the behavior of axon-specific MAPs like Tau. Since our cellular experiments were conducted in neuronal cell models, the quantitative effects reported here may differ in other cell types with distinct tubulin isotype compositions, PTMs landscapes, and MAP expression profiles as highlighted below.

Neurons critically depend on efficient intracellular transport, which is essential for cargo trafficking from the soma to synaptic terminals (via kinesin-mediated anterograde transport) and in the reverse direction (via dynein-mediated retrograde transport). Our data in SH-SY5Y neuroblastoma cells reveal a distinct effect of MT lattice expansion on retrograde transport, particularly in reducing the distance measured by the dynein binding probe, suggesting that PTX may impair the intracellular trafficking. In contrast, kinesin-1-binding probe appears less sensitive to lattice expansion and more influenced by other structural features. Unexpectedly, compound **1b** also reduced both length and speed of kinesin-1 binding probe transport, possibly due to enforced MT homogeneity in cells, where MTs typically display dynamic, asymmetric structural fluctuations.^46^ The similarity of this effect to PTX treatment highlights the complex regulation of kinesin-1 movement by MT lattice features. Differences from our previous results in A549 cells^28^ may arise from differences in the tubulin code, which is known to influence motor interactions with MTs,^47^ as well as differential drug uptake and, drug’s binding affinity to tubulin and MTs. In addition, we have found significant differences with previously reported PTX-induced increases in retrograde mitochondria transport and enhanced anterograde velocity.^2^ This study used lentiviral transduction of Mito-dsRed to label mitochondria, which in combination with differences in cell lines, PTX exposure duration, and dosage could also account for the observed discrepancies. Furthermore, the mobilization of mitochondria is mediated by multiple motors simultaneously. Recent *in vitro* assays demonstrated increased gliding velocities of PTX- and GMPCPP-MTs (expanded lattices) propelled by synchronized teams of kinesin-1 motors.^22^ The mixed results provided by our kinesin-1 single molecule stepping assays (increased velocity on PTX-MTs while decreased on GMPCPP-MTs) indicate that synchronized teams of motors, for example propelling organelle movement *in vivo*, might be affected differently than single kinesin molecules. Follow-up *in vitro* experiments further clarified how distinct structural changes influence kinesin-1 binding and motility: lattice expansion decreases landing rates, while alteration on the PF number, which is a feature already described on axonal MTs,^48^ enhances kinesin-1 affinity. However, when both axial and lateral modifications are present, the effects become more complex. Compared to native-like MTs, PTX-stabilized lattices reduce run length (in line with cellular observations, although velocity is unaffected *in vitro*).

Tau binding to MTs is generally considered not to strongly impact axonal transport rates,^49^ likely due to its “kiss-and-hop” dynamics.^34^ Our results in differentiated PC12 neuronal cells demonstrate that PTX-induced lattice expansion enhances Tau binding to MTs, which in turn has the potential to impair axonal transport.^33,34^ These findings should not be interpreted as evidence that Tau directly mediates taxane-induced peripheral neuropathy. Instead, we show that it is a sensitive neuronal MAP reported, illustrating how MT lattice state modifies MAPs recognition and dynamics. Using a simplified *in vitro* system, we uncovered the structural basis of Tau-MT recognition. We show that Tau envelope formation arises from specific lattice features that promote cooperative Tau assembly once nucleation occurs. Moreover, our data suggest that Tau senses both axial and lateral structural cues, which shape its binding kinetics and, in turn, influence transport regulation and other MAPs interaction.^36–38^ Importantly, Tau preferentially recognizes compressed lattices. While envelope formation is still possible on expanded lattices, its growth rate is markedly slower, consistent with direct competition between PTX and Tau binding.^50^ Taken together, our *in vitro* and cellular findings indicate that on PTX-stabilized MTs, Tau’s initial affinity decreases, but its dwell time once bound increases, likely due to PTX release,^50^ resulting in reduced kiss-and-hop dynamics.

In conclusion, our study underscores the functional relevance of MT heterogeneity as a key determinant of the diverse biological processes mediated by the cytoskeleton. This diversity should be carefully considered in the context of pharmacological intervention. At the same time, we show that compound **1b** preserves the stabilizing activity of PTX on tubulin and MTs, albeit with lower potency, while maintaining a near-native lattice architecture. Taken all of our results together, by establishing that taxane-induced polymerization and lattice remodeling can be uncoupled, we have built a structure–function framework that opens new avenues for the rational design of MT stabilizers with improved biological compatibility.

## Experimental Section

### Protein and Chemicals

Purified calf brain tubulin and chemicals were obtained as previously described.^24^ Tau-mCherry was expressed and purified as described previously.^29^ Human Kif5B-EGFP (amino acids 1-905), containing a C terminal fluorescent-tag followed by a 3C protease cleavage site and a 6 × His-tag was expressed and purified as in^51^ with some modifications. Prior to purification, cells were resuspended in 1:1 ratio of lysis buffer (30 mM HEPES pH 7.5, 300 mM NaCl, 0.5 mM ATP, 5% glycerol, 20 mM imidazole, 0.5 M TCEP) supplemented with 1× Protease inhibitor cocktail and benzonase to the final concentration of 25 units/ml. Cells were homogenized by repeated pipetting. Cells were lysed by spinning for 45 min at 40,000 rpm in 45 Ti fixed-angle rotor (Beckman Coulter) at 4 °C. The supernatant was incubated with pre-equilibrated HisPUR NiNTA agarose resin (Thermo Fisher Scientific) for 1 h in a tightly closed gravity-flow column (Thermo Fisher Scientific) at 4 °C by slowly rotating. Subsequently, the resin was washed once lysis buffer and then with buffer B (30 mM HEPES pH 7.5, 300 mM NaCl, 0.5 mM ATP, 5% glycerol, 30 mM imidazole, 0.5 M TCEP). The protein was eluted from the resin after an overnight cleavage of the 6 × His-tag with 3C HRV protease in lysis buffer at 4 °C. The eluted protein was further purified via size exclusion chromatography on Superdex 200 10/300 GL column equilibrated in exclusion buffer (30 mM HEPES pH 7.5, 300 mM NaCl, 0.5 mM ATP, 5% glycerol, 0.5 M TCEP). Peak fractions were analyzed via SDS-PAGE, pooled and the protein was concentrated with Amicon Ultra-15 Centrifugal Filter tubes (Millipore). Purified proteins were aliquoted, snap-frozen in liquid N_2_, and stored at −80 °C.

Compound concentrations were selected according to the purpose of each assay. For fiber diffraction and *in vitro* reconstitution experiments, concentrations were chosen to promote high taxane-site occupancy and to impose defined and stabilized MT lattice architecture. For cellular imaging and trafficking assays, concentrations were selected on the basis of preliminary stabilization and imaging controls to generate robust MT-stabilized phenotypes without compromising cell viability during the time course of the experiment. These concentrations were not intended to provide equimolar or IC_50_-matched comparisons, nor to establish intrinsic potency or maximal efficacy. Rather, they were used to compare the functional consequences of drug-imposed stabilized MT states.

### Fiber Diffraction

X-ray fiber diffraction data were collected in beamline BL11-NDC-SWEET at ALBA Synchrotron. The energy of the incident photons was 15 keV or equivalently a wavelength, λ, of 0.827 Å.^10^ Purified bovine brain tubulin was diluted to a final concentration of 100 μM in FD buffer (0.08 M Pipes–K, 1 mM EGTA, 0.2 M Tris, 1 mM DTT, 3 mM MgCl_2_ pH 6.8) and incubated on ice for 10 min. Insoluble aggregates were removed by centrifugation at 13,500 rpm for 10 min at 4 °C using Spin-X 0.45 μM cellulose acetate filters (Costar, Corning, USA). Tubulin was then supplemented with 2 mM GTP or 0.5 mM GMPCPP and 200 μM of the tested ligands. All samples were incubated for 30 min at 37 °C to induce the maximum fraction of polymerized tubulin and then, mixed in a 1:1 volume ratio with FD buffer containing 2% methylcellulose (MO512; Sigma-Aldrich). Samples were centrifuged 10 s at 2000 g to eliminate air bubbles and transferred to the space between a mica disc and a ring-shaped coverslip arranged in the shear-flow device.^52^ The device was kept at 37 °C during measurements and the quartz disc rotates at 10 r.p.s to achieve the shear flow alignment. The SAXS diffraction patterns were collected in a Dectris PILATUS3S-1M, with an active area of 168.7 × 179.4 mm^2^, an effective pixel size of 172 × 172 mm^2^ and a dynamic range of 20 bits. Background images were acquired in the same conditions, using PM buffer with methylcellulose without tubulin. Four diffraction images per sample were acquired, each with a 150 s exposure, as higher frame numbers risked radiation damage. For each condition, 8–12 images were collected across 2–3 independent experiments.

#### Data Processing

The whole analyses were performed using custom routines implemented in Python, leveraging libraries such as *NumPy*,^53^ *pandas*, and *SciPy*.^54^ Raw fiber diffraction images were first corrected by subtracting an averaged background image. For each condition, multiple buffer images were normalized and averaged to generate a representative background signal that was subtracted pixel-wise from each sample image to isolate the scattering signal of the sample. This preprocessing step eliminates parasitic scattering contributions and enhances the contrast of the diffractograms. Subsequently, images were subjected to azimuthal integration using the pyFAI library^55^ and a previously calibrated geometry file (poni file), containing detector distance, beam center, and tilt angles, and obtained via standard calibration images with silver behenate. Integration was carried out in specific azimuthal sectors centered around the equatorial and meridional axes. To improve signal quality along the equatorial axis, we also subtracted the residual background scattering from nonoriented species (mainly unassembled tubulin). For doing this, we considered two off-axis regions located at ± 45° relative to the equator because at these areas the scattering from the oriented species is expected to be negligible. Polarization correction and solid angle normalization were applied during the integration. The resulting profiles provided the scattering intensity as a function of the scattering vector *q* ([4π sin­(θ)]/λ), with separate curves for equatorial and meridional directions. Finally, 1D scattering profiles were analyzed to determine main structural parameters of the fiber assemblies. Equatorial profiles were initially fitted using a diffraction model based on previous approaches^56^ of the form *I*(*q*) = *w_n_* · ([*J*_0_(*q*·*r_m_*) · *F*(*U*)]^2^ + *f_n_* · [*J_n_* (*q*·*r_m_*) · *F*(*U*)]^2^) and hence, considering the zeroth- and *n*th-order Bessel functions of the first kind, which represent the two helical scattering components of the MT lattice. However, we found that using *J*_0_ and *J_n_* the fitting was not satisfactory because only applied to minimal regions of the profile. Considering that *J*_01_ is affected by central scattering and *J*_03_ could be convoluted with *J_n_*, we selected *J*_02_ for approaching a new fitting. Because determination of the PF distribution required analysis of higher-order peaks (*J*_03_, *J*_04_, and *J_n_*), the mean PF number was derived from a second fitting of higher-order peaks. For this, we assumed a PF spacing of 5.7 nm^18^ and considered electron microscopy observations that indicate a Gaussian distribution of the PF numbers,^57^ and hence, we modeled a Gaussian centered on the mean. The model was able to reconstruct the scattering profile of MT solutions as a linear combination of the calculated profiles of individual species, suggesting additional information that could be obtained from full radial integration. Meridional peaks were fitted using a four-parameter Lorentzian function to determine the axial repeat distance by fitting the 1 nm^–1^ layer line, *I*(*q*) = *y*_0_ + *a*/[1 + ((*q* – *q*_0_)/*b*)^2^].

### Synthesis of Compounds 1a and 1b

Unless otherwise stated reagents were purchased from general suppliers (Merck, Fluorochem, TCI) and used without further purification. PTX was kindly supplied by Indena s.p.a. (Italy). All solvents were of reagent grade or HPLC grade. All reactions were carried out in oven-dried glassware and dry solvents, under nitrogen atmosphere, and were monitored by glass or aluminum TLC on silica gel (Merck precoated 60F254 plates), with detection by UV light (254 nm), or by TLC stains as potassium permanganate, bromocresol green or panisaldehyde stain. Purification of intermediates and final products was mostly carried out by flash column chromatography, using high purity grade silicagel (Merck grade, pore size 60 Å, 230–400 mesh particle size, Sigma-Aldrich) as stationary phase. Alternatively, purification was performed using Biotage Isolera One system in direct pase using Biotage Sfär Silica D cartridges (4/10/35 g). ^1^H NMR and ^13^C NMR spectra were recorded on a Bruker Avance Spectrometer 400 MHz using commercially available deuterated solvents (chloroform-d, dimethylsulfoxide-d6) at RT. Chemical shifts (δ) are reported in parts per million (ppm) and are reported relative to tetramethylsilane, used as an internal standard. Mass spectrometry (MS) analyses were performed by direct infusion of the samples into an LCQ Fleet ion trap mass spectrometer equipped with an electrospray ionization (ESI) source. MS-SCAN conditions: 50–2000 amu, SCAN-time: 0.2 s. Specific optical rotation, or [α]*D*, was measured on a Jasco P-1030 polarimeter in chloroform. This intrinsic property of chiral compounds is defined by the formula [α]­λ = α/γ*l*, where α is the angle through which plane polarized light is rotated by a compound’s solution of mass concentration γ and path length *l* (in this case *l* is equal to 10 cm, the length of the polarimeter cell). The superscript indicates the Celsius temperature at which the measurement is carried out, while the wavelength of the measurement is reported in the subscript. The letter *D* indicates that the wavelength used corresponds to the sodium *D* line (589 nm). The standard deviation is calculated over 10 measurements. All compounds are >95% pure by HPLC, NMR, MS and optical rotation analyses. A synthetic scheme is presented in Figure S3.

#### 2′-Carboxybenzoylpaclitaxel (7)

Benzyl chloroformate (990 μL, 7.02 mmol) was added in 10 equiv aliquots (165 μL per aliquot) at 10 min intervals to a solution of PTX (100 mg, 0.117 mmol) and dry pyridine (950 μL, 11.71 mmol) in dry CH_2_Cl_2_ (5 mL), at RT and under N_2_ atmosphere. The reaction was stirred for 24 h, after which it was quenched with 2 mL of saturated aqueous NH_4_Cl. After the addition of AcOEt (10 mL), the organic phase was washed with saturated aqueous NH_4_Cl (3 × 5 mL) and brine (2 × 5 mL). The organic layer was then dried over Na_2_SO_4_, and the solvent was evaporated under reduced pressure. The crude product was purified by flash chromatography (silicagel, eluent mixture: 1:1 *n*-hexane/AcOEt) to obtain pure 7 (80.8 mg, 0.082 mmol, 70% yield).

^1^H NMR (400 MHz, CDCl_3_) δ 8.17 (d, *J* = 7.4 Hz, 2H, o-2-BzO), 7.76 (d, *J* = 7.1 Hz, 2H, o-3′-NHBz), 7.68–7.59 (m, 1H, p-2-BzO), 7.56–7.50 (m, 3H, m-2-BzO, p-3′-NHBz), 7.49–7.34 (m, 12H, m-3′-NHBz, o,m,p-2′-CbzO, o,m,p-3′-Ph), 6.98 (d, *J* = 9.3 Hz, 1H, 3′-NH), 6.37–6.28 (m, 2H, 10-H, 13-H), 6.02 (dd, *J* = 9.3, 2.7 Hz, 1H, 3′-H), 5.72 (d, *J* = 7.1 Hz, 1H, 2-H), 5.48 (d, *J* = 2.7 Hz, 1H, 2′-H), 5.26–5.14 (m, 2H, 4′-H), 5.01 (dd, *J* = 9.7, 2.3 Hz, 1H, 5-H), 4.47 (dd, *J* = 10.9, 6.6 Hz, 1H, 7-H), 4.35 (d, *J* = 8.5 Hz, 1H, 20a-H), 4.24 (d, *J* = 8.5 Hz, 1H, 20b-H), 3.85 (d, *J* = 7.0 Hz, 1H, 3-H), 2.59 (ddd, *J* = 14.7, 9.7, 6.5 Hz, 1H, 6a-H), 2.49 (s, 3H, 4-OAc), 2.47–2.41 (m, 1H, 14a-H), 2.26 (s, 3H, 10-OAc), 2.28–2.22 (m, 3H, 14a-H), 1.96 (s, 3H, 18-H), 1.98–1.86 (m, 1H, 6b-H), 1.72 (s, 3H, 19-H), 1.28 (s, 3H, 17-H), 1.17 (s, 3H, 16-H).

^13^C NMR (101 MHz, CDCl_3_) δ 203.97, 171.42, 170.01, 168.01, 167.23, 167.20, 142.89, 133.84, 132.97, 132.17, 130.39, 129.26, 129.06, 128.90, 128.85, 128.66, 128.56, 127.31, 126.74, 84.61, 81.24, 79.39, 77.02, 76.62, 75.76, 75.28, 72.31, 72.23, 70.93, 58.70, 52.91, 45.72, 35.76, 35.68, 27.00, 22.85, 22.32, 20.98, 14.96, 9.76 (detectable peaks).

#### Hexanedioic Acid Mono­(2-(trimethylsilyl)­ethyl) Ester (5a)

Hexanedioic acid (1 g, 6.84 mmol) was added to a mixture of dry CH_2_Cl_2_ and dry pyridine (25 and 2.5 mL respectively, 10:1 ratio) and stirred at RT, under N_2_ atmosphere, until complete dissolution. Then 2- (trimethylsilyl) ethanol (392 μL, 2.74 mmol), EDC·HCl (788 mg, 4.11 mmol) and DMAP (167 mg, 1.39 mmol) were added, and the solution was stirred for 24 h. The reaction mixture was washed with phosphoric acid 10% (2 × 15 mL) and brine (20 mL). The organic phase was then dried over Na_2_SO_4_, and the solvent was evaporated under reduced pressure. The monoprotected product was isolated by flash chromatography (silicagel, eluent mixture: 8:2 *n*-hexane/AcOEt + 1% formic acid) to obtain pure 5a (446 mg, 1.8 1 mmol, 66% yield).

^1^H NMR (400 MHz, CDCl_3_) δ 10.94 (br s, 1H, COOH), 4.09–3.94 (m, 2H, 5-H), 2.29–2.06 (m, 4H, 1-H, 4-H), 1.54 (br s, 4H, 2-H, 3-H), 0. 91–0.76 (m, 2H, 6-H), −0.12 (m, 9H, SiMe3).

^13^C NMR (101 MHz, CDCl_3_) δ 178.87, 173.45, 62.43, 33.83, 33.42, 24.08, 23.89, 17.08, −1.82.

#### Nonanedioic Acid Mono­(2-(trimethylsilyl)­ethyl) Ester (5b)

Nonanedioic acid (1 g, 5.31 mmol) was added to a mixture of dry CH_2_Cl_2_ and dry pyridine (25 and 2.5 mL respectively, 10:1 ratio) and stirred at RT, under N_2_ atmosphere, until complete dissolution. Then 2- (trimethylsilyl) ethanol (304 μL, 2.12 mmol), EDC·HCl (612 mg, 3.19 mmol) and DMAP (130 mg, 1.06 mmol) were added, and the solution was stirred for 24 h. The reaction mixture was washed with phosphoric acid 10% (2 × 15 mL) and brine (20 mL). The organic phase was then dried over Na_2_SO_4_, and the solvent was evaporated under reduced pressure. The monoprotected product was isolated by flash chromatography (silicagel, eluent mixture: 75:25 *n*-hexane/AcOEt + 1% formic acid) to obtain pure 5b (430 mg, 1.49 mmol, 70% yield).

^1^H NMR (400 MHz, CDCl_3_) δ 10.98 (s, 1H, COOH), 4.15–4.05 (m, 2H, 8-H), 2.33–2.16 (m, 4H, 1-H, 7-H), 1.63–1.49 (m, 4H, 2-H, 6-H), 1.32–1.21 (m, 6H, 3-H, 4-H, 5-H), 0.97–0.86 (m, 2H, 9-H), 0.01 (s, 9H, SiMe3).

^13^C NMR (101 MHz, CDCl_3_) δ 179.99, 174.13, 62.51, 34.52, 34.09, 28.97, 28.93, 28.91, 24.94, 24.66, 17.39, −1.43.

#### 2′-O-Cbz-7-O-(1″-O- (2‴-(Trimethylsilyl)­ethyl)­hexanedioyl)-paclitaxel (4a)

A solution of acid 5a (49 mg, 0.2 mmol) in CH_2_Cl_2_ (1 mL) was added to a stirred solution of 2′-O-Cbz-paclitaxel 7 (50 mg, 0.05 mmol) and DMAP (6 mg, 0.05 mmol) in CH_2_Cl_2_ (1 mL), at RT and under N_2_ atmosphere. Then DCC (42 mg, 0.2 mmol) was added, and the reaction mixture was stirred at RT for 18 h. The mixture was diluted with CH_2_Cl_2_ (5 mL) and it was filtered to remove the urea formed as a byproduct. The collected organic phase was washed with saturated aqueous NH_4_Cl (2 × 5 mL) and brine (2 × 5 mL), then it was dried over Na_2_SO_4_, and the solvent was evaporated under reduced pressure. The resulting crude was purified by flash chromatography (silicagel, eluent mixture: 6.5:3.5 *n*-hexane/AcOEt) to obtain compound 4a (55 mg, 0.045 mmol, 90% yield).

^1^H NMR (400 MHz, CDCl_3_) δ 8.p (d, 2H, o-2-BzO), 7.62–7.55 (m, 2H, o-3′-NHBz), 7.51–7.42 (m, 1H, p-2-BzO), 7.41–7.30 (m, 3H, m-2-BzO, p-3′-NHBz), 7.30–7.16 (m, 12H, m-3′-NHBz, o,m,p-2′-CbzO, o,m,p-3′-Ph), 6.78 (d, *J* = 9.3 Hz, 1H, 3′-NH), 6.13 (s, 1H, H-10), 6.16–6.07 (m, 1H, H-13), 5.84 (dd, *J* = 9.3, 2.7 Hz, 1H, 3′-H), 5.55 (d, *J* = 6.9 Hz, 1H, 2-H), 5.45 (dd, *J* = 10.5, 7.0 Hz, 1H, 7-H), 5.32 (d, *J* = 2.7 Hz, 1H, 2′-H), 5.08–4.96 (m, 2H, 4′-H), 4.82 (dd, *J* = 9.6, 1.9 Hz, 1H, 5-H), 4.19 (d, *J* = 8.4 Hz, 1H, 20a-H), 4.05 (d, *J* = 8.4 Hz, 1H, 20b-H), 4.04–3.98 (m, 3H, 5′′-H), 3.81 (d, *J* = 6.9 Hz, 1H, 3-H), 2.46 (ddd, *J* = 14.4, 9.6, 7.1 Hz, 1H, 6a-H), 2.30 (s, 3H, 4-OAc), 2.25 (m, 2H, 14a-H), 2.19–2.10 (m, 4H, 1″-H, 4″-H), 2.07 (m, 14b-H), 2.01 (s, 3H, 10-OAc), 1.85 (s, 3H, 18-H), 1.67 (s, 3H, 19-H), 1.73–1.67 (m, 1H, 6b-H) 1.54–1.44 (m, 4H, 2″-H, 3″-H), 1.07 (s, 3H, 17-H), 1.02 (s, 3H,16-H), 0.89–0.79 (m, 2H, 6″-H), −0.11 (s, 9H, SiMe3).

^13^C NMR (101 MHz, CDCl_3_) δ 201.87, 173.45, 172.17, 169.38, 168.60, 167.77, 167.08, 166.72, 153.82, 140.90, 136.50, 134.12, 133.50, 133.34, 132.36, 131.81, 130.01, 128.97, 128.90, 128.68, 128.54, 128.52, 128.49, 128.34, 128.29, 126.96, 126.40, 83.81, 80.69, 78.46, 76.55, 76.17, 75.02, 74.39, 71.83, 71.02, 70.61, 62.25, 55.85, 52.59, 46.68, 43.09, 35.21, 33.97, 33.45, 33.17, 26.25, 24.13, 23.72, 22.44, 21.09, 20.51, 17.11, 14.31, 10.69, −1.69.

#### Synthesis of 6-O-(2′,3′-dihydroinden-1′-yl)-1-O-(2″-(trimethylsilyl)­ethyl) Hexanedioate (10a)

DMAP (26 mg, 0.21 mmol), EDC·HCl (119 mg, 0.62 mmol), and 1-indanol (87 mg, 0.62 mmol) were added sequentially to a solution of 5a (101.5 mg, 0.41 mmol) in dry CH_2_Cl_2_ and pyridine (18 and 2 mL respectively, 10:1 ratio), at RT and under N_2_ atmosphere. The reaction mixture was stirred for 6 h. When TLC monitoring confirmed reaction completion, HCl 1 M was added (40 mL). The aqueous phase was extracted with CH_2_Cl_2_ (4 × 15 mL), then the collected organic layer was dried over Na_2_SO_4_, and the solvent was evaporated under reduced pressure. The crude oil was purified by Biotage direct phase flash chromatography (eluent mixture: *n*-hexane/AcOEt from 98:2 to 80:20) to afford pure compound 10a (90.9 mg, 0.25 mmol, 61%).

^1^H NMR (400 MHz, CDCl_3_) δ 7.39 (d, *J* = 7.4 Hz, 1H, 10-H), 7.31–7.16 (m, 3H, 13-H, 12-H, 11-H), 6.21 (dd, *J* = 6.9, 3.9 Hz, 1H, 7-H), 4.20–4.11 (m, 2H, H-2), 3.16–3.04 (m, 1H, 9a-H), 2.92–2.81 (m, 1H, 9b-H), 2.56–2.43 (m, 1H, 8a-H), 2.36–2.24 (m, 4H, 3-H, 6-H), 2.13–2.01 (m, 1H, 8b-H), 1.71–1.61 (m, 4H, 4-H, 5-H), 1.02–0.93 (m, 2H, 1-H), 0.04 (s, 9H, -SiMe3).

^13^C NMR (101 MHz, CDCl_3_) δ 173.45, 173.35, 144.39, 141.19, 128.95, 126.76, 125.55, 124.85, 78.31, 62.53, 34.22, 34.14, 32.40, 30.26, 24.55, 24.47, 17.41, −1.42.

#### Hexanedioic Acid Mono­(2′,3′-dihydroinden-1′-yl) Ester (9a)

TBAF 1 M in THF (1 mL, 1 mmol) was added dropwise to a stirred solution of 10a (90 mg, 0.25 mmol) in dry THF (10 mL), at −5 °C and under N_2_ atmosphere. The reaction mixture was warmed up to RT and stirred for 4 h. When TLC monitoring confirmed reaction completion, saturated aqueous NH_4_Cl (15 mL) was added. The aqueous phase was extracted with AcOEt (4 × 15 mL), then the collected organic layer was dried over Na_2_SO_4_ and evaporated under reduced pressure. The crude oil was purified by flash chromatography (silicagel, eluent mixture: 8:2 *n*-hexane/AcOEt + 1% formic acid) to obtain pure 9a (41.3 mg, 0.157 mmol, 63% yield).

^1^H NMR (400 MHz, CDCl_3_) δ 7.38 (d, *J* = 7.4 Hz, 1H, 8-H), 7.37–7.05 (m, 3H, 11-H, 10-H, 9-H), 6.20 (dd, *J* = 6.9, 3.8 Hz, 1H, 5-H), 3.15–3.03 (m, 1H, 7a-H), 2.92–2.80 (m, 1H, 7b-H), 2.55–2.43 (m, 1H, 6a-H), 2.39–2.29 (m, 4H, 1-H, 4-H), 2.18–2.00 (m, 1H, 6b-H), 1.79–1.54 (m, 4H, 2-H, 3-H).

^13^C NMR (101 MHz, CDCl_3_) δ 173.42, 144.50, 141.18, 129.05, 126.84, 125.62, 124.93, 78.46, 34.23, 33.52, 32.44, 30.32, 24.46, 24.19.

#### 2′-O-Cbz-7-O-(6″-O-(2‴,3‴-Dihydroinden-1‴-yl)­hexanedioyl)­paclitaxel (2a)

A solution of compound 9a (28 mg, 0.107 mmol) in CH_2_Cl_2_ (2 mL) was added to a stirred solution of 2′-O-Cbzpaclitaxel 7 (70 mg, 0.071 mmol) and DMAP (8.7 mg, 0.071 mmol) in CH_2_Cl_2_ (3 mL), at RT and under N_2_ atmosphere. Then, DCC (58 mg, 0.284 mmol) was added and the reaction mixture was stirred at RT for 29 h. The mixture was diluted with CH_2_Cl_2_ (8 mL) and filtered on a plug of Celite. The organic phase was washed with saturated aqueous NH_4_Cl (2 × 10 mL) and brine (2 × 10 mL), then it was dried over Na_2_SO_4_, and the solvent was evaporated under reduced pressure. The resulting crude was purified by flash chromatography (silicagel, eluent mixture: 7:3 *n*-hexane/AcOEt) to obtain compound 2a (61.1 mg, 0.050 mmol, 70% yield).

^1^H NMR (400 MHz, CDCl_3_) δ 8.12 (d, *J* = 7.3 Hz, 2H, o-2-BzO), 7.73 (d, *J* = 7.3 Hz, 2H, o-3′-NHBz), 7.64–7.56 (m, 1H p-2-BzO), 7.54–7.44 (m, 3H, m-2-BzO, p-3′-NHBz), 7.43–7.29 (m, 13H, m-3′-NHBz, o,m,p-2′-CbzO, o,m,p-3′-Ph, 8″-H), 7.29–7.18 (m, 3H, 9″-H, 10″-H, 11″-H), 6.95 (d, *J* = 9.3 Hz, 1H, 3′-NH), 6.28–6.20 (m, 2H, 10-H, 13-H), 6.19 (dd, *J* = 7.0, 3.9 Hz, 1H, 5′′-H), 5.97 (dd, *J* = 9.3, 2.5 Hz, 1H, 3′-H), 5.69 (d, *J* = 6.9 Hz, 1H, 2-H), 5.58 (dd, *J* = 10.4, 7.1 Hz, 1H, 7-H), 5.46 (d, *J* = 2.7 Hz, 1H, 2′-H), 5.21–5.10 (m, 2H, 4′-H), 4.95 (d, *J* = 8.9 Hz, 1H, 5-H), 4.32 (d, *J* = 8.4 Hz, 1H, 20a-H), 4.19 (d, *J* = 8.4 Hz, 1H, 20b-H), 3.95 (d, *J* = 6.9 Hz, 1H, 3-H), 3.15–3.03 (m, 1H, 7″a-H), 2.86 (ddd, *J* = 16.0, 8.5, 5.1 Hz, 1H, 7″b-H), 2.59 (ddd, *J* = 14.5, 9.4, 7.4 Hz, 1H, 6a-H), 2.56–2.44 (m, 1H, 6″a-H), 2.44 (s, 3H, 4-OAc), 2.41–2.16 (m, 6H, 14-H, 1″-H, 4″-H), 2.12 (s, 3H, 10-OAc), 2.11–2.00 (m, 2H), 1.99 (s, 3H, 18-H), 1.94–1.82 (m, 1H, 6b-H), 1.80 (s, 3H, 19-H), 1.71–1.57 (m, 4H, 2″-H, 3″-H), 1.20 (s, 3H, 17-H), 1.16 (s, 3H, 16-H).

^13^C NMR (101 MHz, CDCl_3_) δ 202.06, 172.34, 169.59, 168.79, 167.98, 166.99, 141.14, 136.78, 133.72, 133.60, 132.57, 132.00, 130.23, 129.11, 128.90, 128.86, 128.75, 128.71, 128.55, 128.50, 127.17, 126.72, 126.63, 125.53, 124.78, 84.03, 80.92, 78.75, 78.19, 76.81, 76.41, 75.24, 74.62, 72.04, 71.23, 70.82, 56.09, 52.84, 46.91, 43.31, 35.45, 34.23, 33.95, 33.65, 32.36, 30.21, 26.48, 24.39, 23.90, 22.66, 21.30, 20.70, 14.53, 10.90 (detectable peaks).

#### 7-O-(6″-O-(2‴,3‴-Dihydroinden-1‴-yl)­hexanedioyl) Paclitaxel (1a)

Pd/C (10% w/w, 3 mg) was added to a solution of 2a (30 mg, 0.024 mmol) in dry MeOH (2 mL) under hydrogen atmosphere. The reaction mixture was stirred at RT, and it was monitored by TLC until completion (5 h). The reaction mixture was then filtered on a Celite pad, which was washed with MeOH (5 mL ×2). The organic phase was dried with Na_2_SO_4_, and the solvent was evaporated under reduced pressure to afford pure compound 1a in high yield (27.6 mg, 0.0238 mmol, 98% yield).

^1^H NMR (400 MHz, CDCl_3_) δ 8.13 (d, *J* = 7.3 Hz, 2H, o-2-BzO), 7.79 (d, *J* = 7.3 Hz, 2H, o-3′-NHBz), 7.68–7.59 (m, 1H, p-2-BzO), 7.57–7.33 (m, 11H, m-2-BzO, m,p-3′-NHBz, o,m,p-3′-Ph, 8″-H), 7.33–7.19 (m, 4H, 3′- NH, 9″-H, 10″-H, 11″-H), 6.26–6.15 (m, 3H, 10-H, 13-H, 5′′-H), 5.87–5.80 (m, 1H, 3′-H), 5.69 (d, *J* = 6.9 Hz, 1H, 2-H), 5.57 (dd, *J* = 10.3, 7.3 Hz, 1H, 7-H), 4.95 (d, *J* = 9.0 Hz, 1H, 5-H), 4.82 (d, *J* = 2.4 Hz, 1H, 2′-H), 4.33 (d, *J* = 8.4 Hz, 1H, 20a-H), 4.21 (d, *J* = 8.4 Hz, 1H, 20b-H), 3.93 (d, *J* = 6.7 Hz, 1H, 3-H), 3.18–3.06 (m, 1H, 7″a-H), 2.89 (ddd, *J* = 15.9, 8.5, 5.1 Hz, 1H, 7″b-H), 2.65–2.45 (m, 2H, 6a-H, 6″a-H), 2.40 (s, 3H, 4-OAc), 2.38–2.20 (m, 6H, 14-H, 1″-H, 4″-H), 2.16 (s, 3H, 10-OAc), 2.16–2.04 (m, 1H, 6″b-H), 1.88–1.79 (m, 7H, 6b-H, 18-H, 19-H), 1.75–1.57 (m, 4H, 2″-H, 3″-H), 1.21 (s, 3H, 17-H), 1.18 (s, 3H, 16-H).

^13^C NMR (101 MHz, CDCl_3_) δ 202.56, 174.14, 173.19, 173.13, 171.00, 169.57, 169.52, 167.69, 167.54, 157.68, 145.01, 141.80, 141.06, 138.81, 134.43, 133.61, 132.54, 130.84, 129.77, 129.59, 129.53, 129.39, 129.33, 128.89, 127.76, 127.37, 126.16, 125.45, 84.58, 81.72, 79.15, 78.86, 77.11, 75.92, 75.02, 74.04, 72.69, 71.88, 56.84, 55.63, 47.63, 43.90, 36.24, 34.86, 34.52, 34.31, 33.00, 30.86, 27.18, 25.03, 24.55, 23.22, 21.48, 21.38, 15.29, 11.48 (detectable peaks).

MS (ESI): *m*/*z* [M + Na]+ calcd. for C62H67NO17Na: 1120.43, found: 1120.75.

[α]_D_ ^25^ ± SD: −290° ± 6.

SMILES CC1­(C)­[C@@]­([C@@H]­(OC­(C2 = CC = CC = C2)=O)­C3­[C@@]­4­(C)­[C@@H]­(OC­(CCCCC­(OC5C­(C = CC = C6)=C6CC5)=O)=O)­C­[C@@H]­7­[C@]­3­(CO7)­OC­(C)=O)­(O)­C­[C@H]­(OC­([C@H]­(O)­[C@@H]­(NC­(C8 = CC = CC = C8)=O)­C9 = CC = CC = C9)=O)­C­(C)=C1­[C@@H]­(OC­(C)=O)­C4 = O

#### 2′-(Trimethylsilyl)­ethyl (S)-9-((7″-methoxy-1″,2″,3″,4″-tetrahydro naphthalen-2″-yl)­amino)-9-oxononanoate (10b)

DMAP (29.6 mg, 0.243 mmol), EDC·HCl (99.8 mg, 0.52 mmol) and (S)-7-methoxy-2-aminotetralin (92.2 mg, 0.52 mmol) were added sequentially to a solution of 5b (99.5 mg, 0.345 mmol) in dry CH_2_Cl_2_ and pyridine (15 and 1.5 mL respectively, 10:1 ratio), at RT and under N_2_ atmosphere. The reaction mixture was stirred for 4 h. When TLC monitoring confirmed reaction completion, HCl 1 M was added (35 mL). The aqueous phase was extracted with CH_2_Cl_2_ (3 × 15 mL), then the collected organic layer was dried over Na_2_SO_4_, and the solvent was evaporated under reduced pressure. The crude oil was purified by direct phase flash chromatography (silicagel, eluent mixture: 85:25 *n*-hexane/AcOEt + 1% formic acid) to afford pure compound 10b in high yield (153.8 mg, 0.344 mmol, 98%).

^1^H NMR (400 MHz, CDCl_3_) δ 6.96 (d, *J* = 8.4 Hz, 1H, 13-H), 6.67 (dd, *J* = 8.4, 2.7 Hz, 1H, 14-H), 6.54 (d, *J* = 2.7 Hz, 1H, 15-H), 6.32 (d, *J* = 7.9 Hz, 1H, 10-NH), 4.28–4.15 (m, 1H, 10-H), 4.16–4.10 (m, 2H, 2-H), 3.72 (s, 3H, 14-OMe), 3.03 (dd, *J* = 16.4, 5.3 Hz, 1H, 16a-H), 2.86–2.68 (m, 2H, 12-H), 2.61 (dd, *J* = 16.4, 8.5 Hz, 1H, 16b- H), 2.24 (t, *J* = 7.5 Hz, 2H, 3-H), 2.20–2.12 (m, 2H, 9-H), 2.03–1.93 (m, 1H, 11a-H), 1.72 (dtd, *J* = 12.6, 9.1, 6.2 Hz, 1H, 11b-H), 1.65–1.51 (m, 4H, 4-H, 8-H), 1.28 (br s, 6H, 5-H, 6-H, 5-H), 1.01–0.92 (m, 2H, 1-H), 0.03 (s, 9H, SiMe).

^13^C NMR (101 MHz, CDCl_3_) δ 174.59, 173.26, 135.89, 130.37, 128.22, 114.61, 113.20, 63.03, 55.89, 55.88, 45.54, 37.52, 36.63, 35.10, 29.69, 29.61, 29.55, 29.47, 26.83, 26.36, 25.53, 17.99, −1.43

#### (S)-9-((7′-Methoxy-1′,2′,3′,4′-tetrahydronaphthalen-2′-yl)­amino)-9-oxononanoic Acid (9b)

TBAF 1 M in THF (1.117 mL, 1.117 mmol) was added dropwise to a stirred solution of 10b (125 mg, 0.279 mmol) in dry THF (11 mL), at −5 °C and under N_2_ atmosphere. The reaction mixture was then warmed up to RT and stirred for 4 h. When TLC monitoring confirmed reaction completion, saturated aqueous NH_4_Cl (15 mL) was added. The aqueous phase was extracted with AcOEt (3 × 15 mL), then the collected organic layer was dried over Na_2_SO_4_, and the solvent was evaporated under reduced pressure. The crude oil was purified by Biotage direct phase flash chromatography (eluent mixture: *n*-hexane/AcOEt + 1% formic acid from 90:10 to 20:80) to obtain pure 9b (84.6 mg, 0.243 mmol, 87% yield).

^1^H NMR (400 MHz, CDCl_3_) δ 7.01 (d, *J* = 8.4 Hz, 1H, 11-H), 6.71 (dd, *J* = 8.4, 2.7 Hz, 1H, 12-H), 6.59 (d, *J* = 2.7 Hz, 1H, 13-H), 5.49 (d, *J* = 7.8 Hz, 1H, 8-NH), 4.37–4.24 (m, 1H, 8-H), 3.77 (s, 3H, OMe), 3.09 (dd, *J* = 16.4, 5.1 Hz, 1H, 14a-H), 2.90–2.71 (m, 2H, 10-H), 2.61 (dd, *J* = 16.4, 7.6 Hz, 1H, 14b-H), 2.34 (t, *J* = 7.5 Hz, 2H, 1-H), 2.20–2.11 (m, 2H, 7-H), 2.07–1.95 (m, 1H, 9a-H), 1.84–1.71 (m, 1H, 9b-H), 1.68–1.56 (m, 4H, 2-H, 6-H), 1.32 (br s, 6H, 3-H, 4-H, 5-H).

^13^C NMR (101 MHz, CDCl_3_) δ 179.37, 173.75, 158.41, 135.81, 130.40, 128.21, 114.63, 113.25, 55.92, 45.67, 37.48, 36.54, 34.67, 29.63, 29.55, 29.50, 29.39, 26.77, 26.37, 25.32.

#### 2′-O-Cbz-7-O-((S)-9″-(7‴-Methoxy-1‴,2‴,3‴,4‴-tetrahydronaphthalen-2‴-yl)­amino)-9″-oxononanoyl) Paclitaxel (2b)

A solution of compound 9b (30.6 mg, 0.088 mmol) in CH_2_Cl_2_ (1.5 mL) was added to a stirred solution of 2′-OCbz- paclitaxel 7 (58 mg, 0.059 mmol) in CH_2_Cl_2_ (1.5 mL), at RT and under N_2_ atmosphere. Subsequently, DCC (49 mg, 0.236 mmol) and DMAP (10 mg, 0.082 mmol) were added and the reaction mixture was stirred at RT for 26 h. The mixture was diluted with CH_2_Cl_2_ (10 mL) and it was filtered on a plug of Celite. The organic layer was washed with saturated aqueous NH_4_Cl (2 × 10 mL) and brine (2 × 10 mL), then it was dried over Na_2_SO_4_, and the solvent was evaporated under reduced pressure. The resulting crude was purified by Biotage direct phase flash chromatography (eluent mixture: *n*-hexane/AcOEt from 88:12 to 0:100) to obtain compound 2a (67.5 mg, 0.051 mmol, 87% yield).

^1^H NMR (400 MHz, CDCl_3_) δ 8.12 (d, *J* = 7.2 Hz, 2H, o-2-BzO), 7.73 (d, *J* = 7.2 Hz, 2H, o-3′-NHBz), 7.64–7.56 (m, 1H, p-2-BzO), 7.54–7.44 (m, 3H, m-2-BzO, p-3′-NHBz), 7.42–7.28 (m, 12H, m-3′-NHBz, o,m,p-2′-CbzO, o,m,p-3′-Ph), 7.04 (d, *J* = 9.3 Hz, 1H, 3′-NH), 6.99 (d, *J* = 8.6 Hz, 1H, 11″-H), 6.70 (d, *J* = 8.5 Hz, 1H, 12″-H), 6.58 (d, *J* = 2.9 Hz, 1H, 13″-H), 6.31–6.20 (m, 2H, 10-H, 13-H), 5.97 (dd, *J* = 9.3, 2.9 Hz, 1H, 3′-H), 5.68 (dd, *J* = 16.5, 7.5 Hz, 2H, 2-H, 7-H), 5.64–5.54 (m, 1H, 8″-NH), 5.46 (d, *J* = 2.8 Hz, 1H, 2′-H), 5.21–5.10 (m, 2H, 4′-H), 4.95 (d, *J* = 9.3 Hz, 1H, 5-H), 4.32 (d, *J* = 8.4 Hz, 1H, 20a-H), 4.29–4.22 (m, 1H, 8′-H), 4.19 (d, *J* = 8.4 Hz, 1H, 20b- H), 3.95 (d, *J* = 6.9 Hz, 1H, 3-H), 3.74 (s, 3H, OMe), 3.07 (dd, *J* = 16.4, 5.1 Hz, 1H, 14″a-H), 2.86–2.72 (m, 2H, 10″-H), 2.60 (dd, *J* = 16.6, 7.8 Hz, 2H, 14″b-H, 6a-H), 2.44 (s, 3H, 4-OAc), 2.42–2.17 (m, 4H, 14a-H, 1″-H), 2.16–2.09 (m, 5H, 10-OAc, 7″-H), 2.00 (s, 3H, 18-H), 1.95–1.84 (m, 2H, 6b-H, 9″a-H), 1.81 (s, 3H, 19-H), 1.77–1.64 (m, 1H, 9″b-H), 1.63–1.52 (m, 4H, 2″-H, 8″-H), 1.34–1.23 (m, 6H, 3″-H, 4″-H, 5″-H), 1.21 (s, 3H, 17-H), 1.16 (s, 3H, 16-H).

^13^C NMR (101 MHz, DMSO-d6) δ 201.59, 171.86, 171.58, 169.87, 169.12, 168.65, 166.33, 165.21, 157.21, 156.61, 153.87, 139.81, 137.01, 135.94, 134.92, 134.07, 133.60, 132.70, 131.57, 129.77, 129.59, 129.35, 128.80, 128.71, 128.55, 128.36, 128.29, 127.57, 127.41, 127.37, 113.50, 112.15, 82.81, 79.75, 77.15, 76.60, 74.50, 74.05, 71.01, 70.83, 69.81, 55.28, 54.91, 54.08, 47.51, 44.57, 42.91, 35.40, 35.33, 33.33, 31.69, 28.92, 28.49, 28.42, 26.61, 26.20, 25.31, 24.44, 22.51, 21.17, 20.47, 13.92, 10.59 (detectable peaks).

#### 7-O-((S)-9″-(7‴-Methoxy-1‴,2‴,3‴,4‴-tetrahydronaphthalen-2‴-yl)­amino) −9″-oxononanoyl) Paclitaxel (1b)

Pd/C (10% w/w, 4.6 mg) was added to a solution of 2b (57 mg, 0.043 mmol) in dry MeOH (4.25 mL) under hydrogen atmosphere. The reaction mixture was stirred at RT, and it was monitored by TLC until completion (4 h). The reaction mixture was then filtered on a Celite pad, which was washed with MeOH (5 mL x2). The organic phase was dried with Na_2_SO_4_, and the solvent was evaporated under reduced pressure. The crude product was purified by Biotage direct phase flash chromatography (eluent mixture: CH_2_Cl_2_/MeOH from 100:0 to 96:4) to afford pure compound 1b (27.6 mg, 0.0238 mmol, 45% yield).

^1^H NMR (400 MHz, CDCl_3_) δ 8.12 (d, *J* = 7.1 Hz, 2H, o-2-BzO), 7.78 (d, *J* = 7.1 Hz, 2H, o-3′-NHBz), 7.66–7.58 (m, 1H, p-2-BzO), 7.55–7.31 (m, 10H, m-2-BzO, m,p-3′-NHBz, o,m,p-3′-Ph), 7.24 (d, *J* = 9.0 Hz, 1H, 3′-NH), 7.01 (d, *J* = 8.4 Hz, 1H, 11″-H), 6.71 (dd, *J* = 8.4, 2.9 Hz, 1H, 12″-H), 6.60 (d, *J* = 2.8 Hz, 1H, 13″-H), 6.24 (s, 1H, 10-H), 6.17 (t, *J* = 8.2 Hz, 1H, 13-H), 5.81 (dd, *J* = 9.0, 2.7 Hz, 1H, 3′-H), 5.67 (d, *J* = 6.9 Hz, 1H, 2-H), 5.55 (dd, *J* = 10.6, 7.2 Hz, 2H, 7-H, 8″-NH), 4.94 (d, *J* = 7.6 Hz, 1H, 5-H), 4.79 (d, *J* = 2.6 Hz, 1H, 2′-H), 4.35–4.23 (m, 2H, 20a-H, 8″-H), 4.19 (d, *J* = 8.5 Hz, 1H, 20b-H), 3.92 (d, *J* = 6.9 Hz, 1H, 3-H), 3.77 (d, *J* = 2.6 Hz, 3H, OMe), 3.09 (dd, *J* = 16.5, 5.3 Hz, 1H, 14″a-H), 2.89–2.73 (m, 2H, 10″-H), 2.66–2.53 (m, 2H, 14″b-H, 6a-H), 2.38 (s, 3H, 4-OAc), 2.36–2.29 (m, 3H, 14a-H, 1″-H), 2.29–2.17 (m, 1H, 14b-H), 2.15 (d, *J* = 6.9 Hz, 5H, 10-OAc, 7″-Hp), 2.02–1.85 (m, 1H, 9″a-H), 1.82 (d, J = 8.0 Hz, 7H, 18-H, 19-H, 6b-H), 1.80–1.65 (m, 1H, 9″b-H), 1.59 (d, *J* = 13.3 Hz, 4H, 2″-H, 8″-H), 1.34–1.22 (m, 6H, 3″-H, 4″-H, 5″-H), 1.20 (s, 3H, 17-H), 1.17 (s, 3H, 16-H).

^13^C NMR (101 MHz, CDCl_3_) δ 202.08, 173.04, 172.78, 172.53, 170.43, 168.96, 167.09, 167.02, 157.85, 140.52, 138.28, 135.37, 133.90, 133.09, 132.01, 130.30, 129.87, 129.23, 129.06, 128.86, 128.80, 128.37, 127.72, 127.22, 114.07, 112.71, 84.06, 81.17, 78.62, 76.59, 75.40, 74.47, 73.47, 72.15, 71.25, 56.34, 55.39, 55.08, 47.11, 45.01, 43.37, 37.07, 36.10, 35.71, 34.17, 33.99, 33.61, 29.16, 29.01, 28.93, 26.66, 25.71, 25.03, 24.51,22.67, 20.92, 20.88, 14.76, 10.96.

MS (ESI): *m*/*z* [M + Na]+ calcd. for C67H78N2O17Na: 1205.52, found: 1205.44.

[α]_D_ ^25^ ± SD: −235° ± 5.

SMILES

CC1­(C)­[C@@]­([C@@H]­(OC­(C2 = CC = CC = C2)=O)­C3­[C@@]­4­(C)­[C@@H]­(OC­(CCCCCCCC­(N­[C@@H]­5CC­(C = C­(OC)C = C6)=C6CC5)=O)=O)­C­[C@@H]­7­[C@]­3­(CO7)­OC­(C)=O)­(O)­C­[C@H]­(OC­([C@H]­(O)­[C@@H]­(NC­(C8 = CC = CC = C8)=O)­C9 = CC = CC = C9)=O)­C­(C)=C1­[C@@H]­(OC­(C)=O)­C4 = O

### Cell Biology

Human NSCLC carcinoma-derived cells A549, human cervical carcinoma HeLa, HeLaβIII (MDR overexpressing βIII tubulin isotype), Kb-3.1 (HeLa subclone), Kb-V1 (MDR overexpressing PgP) and human SH-SY5Y neuroblastoma cell lines were cultured at 37 °C in DMEM supplemented with 10% fetal calf serum, 2 mM l-glutamine, 1 mM sodium pyruvate, 40 μg/mL gentamycin, 100 IU/ml penicillin and 100 μg/mL streptomycin in a 5% CO_2_ air atmosphere.

Indirect immunofluorescence images were obtained using A549 cells plated at a density of 35,000 cells/well onto 18 mm round coverslips, cultured overnight, and treated with increasing amounts of compounds **1a** (200 nM to 8 μM), **1b** (100 nM to 4 μM), PTX (100 nM), or the drug vehicle (DMSO) for 24 h. DMSO was always less than 0.5% v/v. PEG and Triton X-100 were applied to cells prior the fixation with 3.7% formaldehyde, as previously described.^58^ Cells were incubated with a DM1A mouse monoclonal antibody (Sigma-Aldrich) reacting with α-tubulin. After that, samples were washed and incubated with AF488 goat antimouse polyclonal antibody (ThermoFisher), and 3 μM DAPI (Merk) was added for staining the DNA. Images were taken using a Leica DM 6000 B epifluorescence microscope employing a 100X objective with a N.A. of 1.46. Images of α-tubulin and DNA were usually taken at slightly different but close z planes in order to improve the clarity of the focus for each individual image. Images were recorded using a Leica DFC360 FX CCD camera.

Antiproliferative assays were performed as described.^23^ Briefly, the MTT viability assay was used for the determination of the cytotoxicity of compounds **1a**, **1b** and PTX. A549, HeLa, HeLaβIII, Kb-3.1, and Kb-V cells were seeded at densities of 65,000 cells/well (A549, HeLa, Kb-3.1) or 100,000 cells/well (HeLaβIII, Kb-V1), and were grown for 24 h at 37 °C and 5% CO_2_. Then, increasing concentrations of drugs were added and incubated for 48 h prior to the reaction with the MTT solution. IC50 values were obtained by fitting the experimental data (absorbance at 570/690 nm) to a four-parameter logistic curve using SigmaPlot 14.5 software package (Systat Software, Inc., San Jose, CA, USA) and IC50 values were expressed as mean ± SEM from three independent experiments, each performed in duplicate.

### Kinesin and Dynein Intracellular Tracking in SH-SY5Y Cells

Cy5-KBP and Cy5-DBP tracking peptides were synthesized at CIB-Margarita Salas (CSIC), following a published protocol.^28^ Briefly, the peptide chains were assembled via solid phase peptide synthesis (SPPS) using fluorenylmethyloxycarbonyl (Fmoc) as N-terminal protecting group and, subsequently labeled with the organic dye cyanine 5 (Cy5) via a maleimide–thiol reaction between the N-terminal cysteine residues of the peptides and the commercially available Cy5-maleimide. The final peptides were purified via reverse phase HPLC, dissolved in DMSO to a final 2.5 mM concentration and stored in small aliquots at −20 °C. SH-SY5Y cells were seeded into μ-Slide 8-wells ibiTreat plates (ibidi, flat polymer coverslip-bottom, tissue culture treated) at a concentration of 35,000 cells/well and incubated for 48 h at 37 °C and 5% CO_2_. Solutions of the tested compounds at the desired concentrations (1 μM for PTX and FTX2, 2.5 μM for **1a** and **1b**) with and without 2.5 μM Cy5-KBP or Cy5-DBP were prepared in Gibco DMEM without phenol red, previously supplemented with 10% v/v fetal calf serum, 2 mM l-glutamine, 1 mM sodium pyruvate, 40 μg/mL gentamycin, 100 IU/mL penicillin and 100 μg/mL streptomycin, and incubated 20 min at 37 °C and 5% CO_2_. After this time, the culture medium was replaced by the drug working solution containing either Cy5-KBP or Cy5-DBP, and the plate was incubated for an additional 15 min at 37 °C and 5% CO_2_. Then, the culture medium was replaced with drug working solution without labeled peptide and the cells were imaged using a confocal laser scanning microscope Leica TCS SP8 with a 63x oil immersion objective that included a humidified incubation chamber, a CO_2_ controller and a heating unit. Cy5 dye was excited at 635 nm and the fluorescence emission was collected at 645–690 nm. Images were recorded every 1.793 s for 3 min as 2-layer-z-stacks. For each condition, two to three different fields were imaged in two separate experiments.

Single-peptide trajectories were tracked with the TrackMate plugin from the Fiji package software (https://imagej.net/TrackMate). Particles with an estimated blob diameter of 1–1.5 μm were detected using the Laplacian of Gaussian (LoG) detector and linked with the Linear motion LAP tracker. Only spots that were at a maximum distance of 2 μm were linked. Trajectories with less than 6 spots were discarded since they were probably artifactual, and track mean displacement and track mean velocity were calculated and normalized to control cells. Data were further analyzed with MATLAB to perform a Mean Square Displacement (MSD) analysis to determine the mode of displacement of the peptides followed over time.^59^ Briefly, for each trajectory the MSD function was calculated and its log–log representation fitted with a linear function (*log*(*MSD*(τ)) = 2 x *log*(τ) + *C*). MSD curves with an R^2^ coefficient <0.8 were removed. The slope (α) of each MSD curve determines the motion type of each particle^60^ and particles were classified into: constrained particles (CM) α < 1, Brownian particles (BM) α = 1, and transported particles (TM) α > 1. Two independent experiments were performed using three biological replicates (N = 6). Statistical analyses of normalized track mean displacement and track mean velocity were performed using an unpaired *t* test with Welch’s correction in GraphPad Prism 8 software. Statistical significance was set at **** *p* < 0.0001, *** *p* < 0.001, ** *p* < 0.01, and * *p* < 0.05.

### PC12 Culture and Fluorescence Decay After Photoactivation (FDAP)

PC12 cells (originally obtained from J.A. Wagner, Harvard Medical School) were cultured in serum-DMEM (DMEM supplemented with 10% fetal bovine serum and antibiotics (100 U/ml penicillin and 100 μg/mL streptomycin)) at 37 °C with 10% CO_2_ in a humidified incubator. For live cell imaging, cells were transfected with the respective pRc/CMV expression vector using Lipofectamine 2000 (Thermo-Fisher Scientific, USA) as previously described.^61^ FDAP experiments were performed essentially as previously described.^62^ Briefly, cells expressing PAGFP-tagged Tau441 were plated on 35 mm glass-bottom culture dishes (MatTek, USA), transfected, and neuronally differentiated by medium exchange to serum-reduced DMEM containing 100 ng/mL 7S mouse NGF. After 3 days, the medium was exchanged to serum-reduced DMEM without phenol red with NGF, and the respective compound (or DMSO for carrier control) were added at the desired concentration. After 20 h, live cell imaging was performed using a laser scanning microscope (Nikon Eclipse Ti2-) equipped with a LU-N4 laser unit with 488 and 405 nm lasers and a Fluor 60× ultraviolet-corrected objective lens (NA 1.4) and a C2+scanner enclosed in an incubation chamber at 37 °C and 5% CO_2_. Photoactivation was performed with a 405 nm laser using the microscope software (NIS-Elements version AR 5.02.03). The following steps were performed in a photoactivation experiment. A transfected cell with a suitable process (minimum length 30 μm) was selected in the field of view (50 μm × 50 μm) and a preactivation image was saved at 488 nm excitation. The scan window was reduced to a 6-μm diameter activation spot (corresponding to 130 pixels), and photoactivation was performed in the center of the neurite using the 405 nm blue diode (laser intensity 1.0%; corresponding to an output of 30 μW) with two scans at a pixel dwell time of 4.08 μs (corresponding to a total activation time at the spot of 1.1 ms). The scan window was enlarged to the initial size, and the first scan was performed after 1 s with the 488 nm laser at the lowest practical laser intensity. Subsequent images were acquired at a rate of 1 frame/s. 112 images with a resolution of 256 × 256 pixels were collected per activated neurite. To ensure that the imaging conditions did not affect cell viability and subsequent evaluation, neurites were excluded from analysis if they showed retraction or drifted out of focus. Effective diffusion constants were determined by fitting the fluorescence decay data from the photoactivation experiments using a one-dimensional diffusion model function for FDAP according to.^35^ All data sets were tested for normality using the D’Agostino-Pearson and Shapiro-Wilk tests. Statistical outliers were identified using the ROUT method. Equality was assessed using a F-test to compare variances. To compare different concentrations, statistically significant differences from control were determined by a one-way ANOVA with Dunnett’s post hoc test. All statistical analyses were performed using the GraphPad Prism program.

### Biochemistry

Tubulin polymerization assays were performed in PEDTA (10 mM NaP_i_, 1 mM EDTA, 0.1 mM GTP pH 7.0) or GAB (10 mM NaP_i_, 3.4 M glycerol, 1 mM EGTA, 0.1 mM GTP pH 6.7) buffers supplemented with 0.9 mM GTP and 4 mM MgCl_2_. Assembly time courses of 25 μM tubulin at 37 °C were recorded in an Appliskan (ThermoFisher) by measuring absorbance at 350 nm in the presence of 35 μM of compounds **1a, 1b**, PTX, FTX2 or DMSO (as control reaction).

The equilibrium binding constant of **1a** and **1b** to the taxane site of assembled MTs were determined by the displacement of FTX2 from the taxane site in cross-linked MTs.^63^ The displacement isotherm of each ligand was measured at least three times in two different plates with a fluorescence polarization microplate reader, as described.^64^ A mixture of 50 nM taxane binding sites and 50 nM FTX2 in GAB buffer was sampled in a 96-well plate. Increasing concentrations (12.5 nM–25 μM) of the competitor ligands (**1a** or **1b**) were added to the wells. After mixing the samples for 10 min by shaking at 250 rpm, they were incubated 20 min at the desired temperatures (26, 35 or 42 °C) and their anisotropies were measured using a Spark Tecan plater reader in fluorescence polarization at emission of 535 nm and excitation of 485 nm. A sample containing 50 nM taxane binding sites was employed as blank, and a 50 nM solution of FTX2 in GAB, 0.1 mM GTP was employed as anisotropy standard. G factor was calculated as a mean over several cells in different conditions. The molar saturation fraction, *X*_*b*_, of the probe for each competitor concentration is calculated from the polarization values measured, *P*. Measurements of the polarization of FTX2 completely displaced (*P*_*0*_) and, in absence of the competitor ligand (*P*_sat_), are required for data processing. The binding of FTX2 in the presence of the competitor ligand (*X*_*b*_ = (*P–P*_*0*_/*P*_*sat*_ – *P*_*0*_) · *X*_sat_) was calculated and best fitted to the equilibrium binding constant of the competitor assuming unitary stoichiometry and the binding constant of the reference ligand (FTX2) with a PC program (Equigra v5).^63^

Once the equilibrium binding constants are measured at different temperatures, the standard free energy of binding at a given temperature is calculated from the binding constant as Δ*G*_0app_ = −RT Ln *K*_a_, the standard enthalpy of the binding reaction is calculated from the representation of Ln *K*_a_ versus 1/T (Van’t Hoff representation), and the slope of this representation multiplied by −*R* is equal to −Δ*H*_0app_. The standard entropy of binding to Δ*S*_0app_ is calculated from the slope of the linear regression of Δ*G*_0app_ versus *T*.

### Computational Model Building

*Ab initio* geometry optimization and derivation of atom-centered RESP charges^65^ for FTX2 and **1b** were accomplished using the density functional tight-binding method, a 6–31G­(d,p) basis set, and the IEF-SCRF continuum solvent model^66^ for water, as implemented in program Gaussian 09 (Revision D.01).^67^ The conformational preferences of FTX2 and **1b** in aqueous solution were studied upon immersion of each ligand in a cubic box of TIP3P water molecules and simulation at 300 K using molecular dynamics (MD), as performed earlier for PTX and other taxanes.^27^ Bonded and nonbonded parameters for the ligands studied were obtained from the general AMBER force field *gaff2*.^68^

Our starting structure of a straight PF segment consisting of three concatenated α:β dimers (α1:β1-α2:β2-α3:β3) has already been reported in detail.^12^ Missing residues 39–48 in the four α subunits were grafted from AlphaFold model AF–P81947-F1-v4 for completeness. For consistency with the Protein Data Bank, residue numbering and secondary structure assignment herein follow the α-tubulin-based definitions.^69^ The GTP and GDP molecules in the nucleotide-binding sites of α- and β-tubulin, respectively, were kept, together with their coordinated Mg^2+^ ions and hydrating water molecules. The taxanes studied were best-fit superimposed onto the structure of bound PTX, as reported earlier for similar molecules,^12^ to yield the corresponding complexes, which were simulated in explicit solvent at 300 K using the AMBER 18 implementation of the *pmemd.cuda_SPFP* engine and the *ff14SB* force field^70^ for protein atoms and TIP3P water molecules, as previously described.

For the ligand-bound macromolecular assemblies representing three parallel short PFs, each consisting of two concatenated head-to-tail dimers (i.e., (α1:β1-α2:β2-α3:β3)/(α1′:β1′-α2′:β2′-α3′:β3′)/(α1″:β1″-α2″:β2″-α3″:β3″)), use was made of PDB entries 6DPU and 6DPV,^71^ which depict a stretch of an expanded or compacted 14-PF MT, respectively, and 3JAK,^72^ which corresponds to a symmetrized reconstruction of a compacted 13-PF MT. The molecular graphics program PyMOL (v. 2.6, Schrödinger) was employed for molecular visualization and editing. The Cα trace of PDB entry 3JAK was also used as input to either the PyANM plugin for PyMOL (https://pymolwiki.org/index.php/PyANM) or the elNémo web server^73^ to build an anisotropic elastic network model^74^ for normal-mode analysis (NMA) using the default values for cutoff (12 Å), force constant (1.0 kcal mol^–1^·Å^–2^) and maximum displacement (amplitude). The NMA allowed us to characterize the first or second, depending on the method, nontrivial mode as that involved in the [low-frequency/large-amplitude] slow bending motion that drives the cooperative structural fluctuations leading to PF number variation. By saving the coordinates of the model corresponding to the maximum amplitude we obtained a suitable template for an [(α1:β1-α2:β2-α3:β3)/(α1′:β1′-α2′:β2′-α3′:β3′)/(α1″:β1″-α2″:β2″-α3″:β3″)] MT patch with a slightly higher degree of curvature (*i.e*., a smaller radius). Six copies of the refined complex were generated in PyMOL. The (α1:β1-α2:β2-α3:β3) stretch of the first copy was then best-fit superimposed onto the (α1″:β1″-α2″:β2″-α3″:β3″) stretch of the harmonically deformed model. Thereafter, the (α1:β1-α2:β2-α3:β3) stretch of each remaining copy was best-fit superimposed onto the (α1″:β1″-α2″:β2″-α3″:β3″) stretch of the preceding model. This process led quite naturally to formation of a tubular structure consisting of 12 straight PFs (Figure S4D) with one PTX molecule bound in the taxane site of every β subunit. Lastly, the macromolecular ensemble was cleaned up to produce an all-atom model of the MT lattice by (i) removing the redundant subunit copies used for the superposition, (ii) renumbering chains, and (iii) carrying out an energy minimization. For each system studied, the interdimer space was first explored in a minimalist complex (β1:α2) in which all Cα atoms – except those in the more flexible and less well-defined loop regions– were positionally restrained by means of a weak harmonic force constant (2.0 kcal mol^–1^·Å^–2^) to preserve the gross characteristics of each MT subtype. The equilibrated system was then subjected to a simulated annealing procedure, by gradually decreasing the temperature from 300 to 100 K under the same conditions, and thereafter to energy minimization in the absence of any restraints, as reported earlier.^12^ The resulting optimized complex representing the interdimer interface was then replicated five times and one copy was superimposed onto the respective pairs in the 2 × 3 patch of tubulin dimers to produce the corresponding MT piece made up of three PFs, each consisting of two concatenated α:β dimers. Thus, six replicas of each bound ligand and ligand-binding site were sampled simultaneously. For these MD simulations, the GTP:Mg^2+^ bound in α-tubulin was kept whereas GDP:Mg^2+^ was placed in all β-tubulin subunits; in all cases, extra care was taken to keep the distinct hydration shells around the bound cations, as reported in the high-resolution X-ray crystal structures deposited with PDB codes 4I4T and 8BDE.

By copying this 3-PF MT piece and superimposing one lateral PF onto another, one complete turn of a MT was generated (Figure S4D). Since we used three different templates (6DPU, 6DPV and 3JAK), we could build three short optimized MTs with distinct properties that were then subjected to an additional energy refinement using the generalized Born model for the bulk solvent,^75^ as implemented in the *pmemd.cuda_SPFP* engine of AMBER. By following this hybrid approach of combining explicit and implicit solvent models within the same simulation, the computational efficiency was increased by more than 100-fold relative to comparable simulations using tens of thousands of water molecules. The atomic models of an expanded PTX-bound 12-PF MT (Model Archive accession code ma-rkctk), a compacted FTX2-bound 14-PF MT and a compacted **1b**-bound 13-PF MT have been deposited in the ModelArchive database^76^ with accession codes ma-ayvpr and ma-galf8, respectively.

### TIRF Microscopy

Total Internal Reflection Fluorescence microscopy experiments were performed on an inverted widefield microscope Nikon-Ti2 E, equipped with a motorized XY-stage, a perfect focus system, Apo TIRF 60× oil NA 1.49 objective and PRIME BSY (Teledyne Photometrics) camera. MTs were visualized using Interference Reflection microscopy (IRM) and fluorescently labeled proteins were visualized sequentially by switching between microscope filter cubes for GFP and mCherry channels. The microscopes were controlled using NIS Elements software (Nikon). All experiments were conducted at RT.

Microscopy chambers were prepared from two sandwiched silanized glass coverslips (22 × 22 mm^2^ and 18 × 18 mm^2^) attached through parafilm strips (heated for 15–20 s at 60 °C and gently pressed together) to form ∼2.5 mm wide parallel flow channels. Prior to the chamber assembly, the coverslips were washed in 3 M KOH, cleaned using Zepto plasma cleaner (Diener electronic) and silanized with hexamethyldisilazane. Flow channels were first incubated with 40 μgml^–1^ antibiotin antibodies (Sigma, B3640) in PBS for 10 min, followed by incubation with 1% Pluronic F127 (Sigma-Aldrich, P2443) in PBS for at least 1 h. F127 was washed with BRB80 (80 mM PIPES, 1 mM EGTA, 1 mM MgCl_2_, pH 6.9).

Porcine brain tubulin was isolated using the high-molarity PIPES procedure^77^ then mixed with Biotin labeled tubulin (Cytoskeleton Inc., T333P) in 1:49 ratio. All MTs were polymerized using 2% biotin-labeled tubulin. PTX postassembly stabilized MTs (GTP polymerized, then PTX stabilized, stored and imaged in presence of PTX) were polymerized from 4 mg mL^–1^ biotinylated tubulin for 30 min at 37 °C in BRB80 supplemented with 4 mM MgCl_2_, 5% DMSO, and 1 mM GTP (Jena Bioscience, NU-1012). Polymerized MTs were centrifuged for 30 min at 18,000*g* in a Microfuge 18 Centrifuge (Beckman Coulter’s). The pellet was resuspended and kept in BRB80 supplemented with 10 μM PTX. Drug stabilized preassembly MTs (polymerized in the presence of drugs and GTP, stored and imaged in presence of drug) were polymerized from 4 mg mL^–1^ biotinylated tubulin for 30 min at 37 °C in BRB80 supplemented with 4 mM MgCl_2_, 40 μM drug of interest in 5% DMSO, and 1 mM GTP. Polymerized MTs were centrifuged for 30 min at 18,000*g* in a Microfuge 18 Centrifuge (Beckman Coulter’s). The pellet was resuspended and kept in BRB80 supplemented with 10 μM PTX. GMPCPP stabilized MTs (GMPCPP polymerized) were assembled from 4 mg mL^–1^ biotinylated tubulin for 2 h at 37 °C in BRB80 supplemented with 1 mM MgCl_2_ and 1 mM GMPCPP (Jena Bioscience, NU-405). Polymerized MTs were centrifuged for 30 min at 18,000*g* in a Microfuge 18 Centrifuge (Beckman Coulter’s) and pellets were resuspended and kept in BRB80.

Stabilized MTs were flushed into the channel and incubated for 15–30 s to allow for surface attachment. Unbound MTs were removed by washing with BRB80 supplemented with 10 μM of drug. Prior the experiment, the solution was exchanged by motility buffer, MB (BRB80 containing 10 mM DTT, 20 mM d-glucose, 0.1% Tween-20, 0.5 mg/mL casein, 1 mM Mg-ATP, 0.02 mg/mL catalase, 0.22 mg/mL glucose oxidase, 10 μM drug). Subsequently, a solution containing either 1.08 nM Kif5b-EGFP or 10/40 nM mCherry-Tau in MB was introduced into the chamber.

To assess kinesin-EGFP motility along MTs, an IRM image (50 ms exposure) was acquired followed by a 1 min video using 488 nm laser excitation (20% laser intensity, 300 ms exposure) with no delay. Two to three videos were recorded from different fields within the same channel and at least 2 channels were measure for each experimental condition. The same experiment was done in 2 to 3 different days giving an n between 8 and 18.

To monitor Tau-mCherry binding to MTs, 4 min videos were acquired with image acquisition every 7 s. Two imaging channels were recorded simultaneously, alternating between IRM (50 ms exposure) and 561 nm field (30% intensity, 200 ms exposure). In the first experiment, 10 nM Tau-mCherry was injected immediately after the first frame was acquired. Similarly, a second video of the same area was recorded adding an additional 40 nM Tau-mCherry.

#### Image Analysis

All data was collected from at least two independent trials. Unless otherwise stated, all data were analyzed manually using Fiji (ImageJ); graphs and statistical analyses were created and performed using GraphPad Prism v.8.0.1. Graphs represent data means, and the error bars represent the standard errors of mean (SEM). Background fluorescence was subtracted from all intensity measurements. For each condition, analyses were performed on 12 randomly selected, in-focus MTs per channel, being identified with IRM. For kinesin motility analysis, MTs were selected in the IRM channel by drawing segmented lines 5 pixels in width. Kymographs were generated using the *KymographBuilder* plugin. Individual motor traces were manually selected as straight lines (1 pixel thick) on the kymographs. From these traces, run length (*x*-axis), interaction time (*y*-axis), and velocity (calculated as the ratio of both) were determined using the Multiple Velocity Measurement Tool macro that applies to all ROIs in the ROI Manager of Fiji. Finally, landing rate was assessed by quantifying the number of kinesins bound along each MT and normalizing this value to the total MT length (expressed in μm) and time. To quantify the fraction of the MT surface covered by Tau envelopes, a segmented line with a fixed width of 5 px (0.36 μm) was manually traced along either the envelope-covered regions or the full length of individual MTs. The total length of all envelope segments was then divided by the corresponding MT length to yield the percentage of coverage. This analysis was conducted at multiple time frames: four time points (12 s, 1, 2, and 4 min) in the video recorded under 10 nM Tau-mCherry, and three time points (12 s, 2 and 4 min) for the 40 nM condition. To calculate the fluorescence intensity between envelope-covered and uncovered regions, the intensity of the manually selected fragments was measured. The mean fluorescence intensity of each ROI was calculated by averaging the pixel intensity values weighted by the corresponding area. For each MT, the mean intensity of envelope-covered regions was divided by the mean intensity of uncovered regions, which was also determined by drawing linear ROIs (5 px in width) on noncovered segments of the same MT. To compare the amount of Tau bound per micron of MT across different structural conditions, a segmented line (5 px wide) was drawn along the entire length of each MT. The integrated fluorescence intensity (IntDen) was measured, and this value was normalized to the background and divided by the corresponding MT length (in μm). This analysis was performed at three time points (12 s, 2 and 4 min) in both sequential videos. To calculate the initial velocity of Tau coverage, the length (in μm) of MT segments covered by Tau within the first 12 s following the injection of 10 nM Tau-mCherry was measured and normalized to the total length of the corresponding MT.

## Supplementary Material





























































## Notes

Shear-flow aligned fiber diffraction images; fiber diffraction profiles of GMPCPP assembled MTs; synthetic scheme of compounds **1a** and **1b**; computational analysis of FTX2, compound **1b**, and PTX; table of normalized motor probes to control experiments; and table of fiber diffraction data analysis under assembly TIRF conditions (PDF)
HPLC traces lead compounds (PDF)
Molecular formula strings (CSV)
**Movie S1**: Normal mode analysis of a cryoEM patch of 3 PFs with 2 tubulin dimers each (MP4)
**Movie S2:** Movement of Cy5-DBP in SH-SY5Y treated with DMSO (AVI)
**Movie S3:** Movement of Cy5-DBP in SH-SY5Y treated with PTX (AVI)
**Movie S4:** Movement of Cy5-DBP in SH-SY5Y treated with FTX2 (AVI)
**Movie S5:** Movement of Cy5-DBP in SH-SY5Y treated with **1a** (AVI)
**Movie S6**: Movement of Cy5-DBP in SH-SY5Y treated with **1b** (AVI)
**Movie S7:** Movement of Cy5-KBP in SH-SY5Y treated with DMSO (AVI)
**Movie S8:** Movement of Cy5-KBP in SH-SY5Y treated with PTX (AVI)
**Movie S9:** Movement of Cy5-KBP in SH-SY5Y treated with FTX2 (AVI)
**Movie S10:** Movement of Cy5-KBP in SH-SY5Y treated with **1a** (AVI)
**Movie S11:** Movement of Cy5-KBP in SH-SY5Y treated with **1b** (AVI)
**Movie S12**: Movement of Kif5B-EGFP along GMPCPP stabilized MTs (AVI)
**Movie S13**: Movement of Kif5B-EGFP along PTX stabilized MTs (AVI)
**Movie S14**: Movement of Kif5B-EGFP along **1a** stabilized MTs (AVI)
**Movie S15**: Movement of Kif5B-EGFP along **1b** stabilized MTs (AVI)
**Movie S16**: Binding of 10 nM Tau-mCherry along GMPCPP stabilized MTs (AVI)
**Movie S17**: Binding of 40 nM Tau-mCherry along GMPCPP stabilized MTs (AVI)
**Movie S18**: Binding of 10 nM Tau-mCherry along PTXpre stabilized MTs (AVI)
**Movie S19**: Binding of 40 nM Tau-mCherry along PTXpre stabilized MTs (AVI)
**Movie S20**: Binding of 10 nM Tau-mCherry along PTXpost stabilized MTs (AVI)
**Movie S21**: Binding of 40 nM Tau-mCherry along PTXpost stabilized MTs (AVI)
**Movie S22**: Binding of 10 nM Tau-mCherry along FTX2 stabilized MTs (AVI)
**Movie S23**: Binding of 40 nM Tau-mCherry along FTX2 stabilized MTs (AVI)
**Movie S24**: Binding of 10 nM Tau-mCherry along **1a** stabilized MTs (AVI)
**Movie S25**: Binding of 40 nM Tau-mCherry along **1a** stabilized MTs (AVI)
**Movie S26**: Binding of 10 nM Tau-mCherry along **1b** stabilized MTs (AVI)
**Movie S27**: Binding of 40 nM Tau-mCherry along **1b** stabilized MTs) (AVI)

## ◆

Laboratory of Biomolecular Research, Division of Biology and Chemistry, Paul Scherrer Institut, Villigen, Switzerland

## Abbreviations Used

2AP: 2-debenzoyl-2-(*m*-aminobenzoyl)­paclitaxel
3AP: 3′-*N*-m-aminobemzamido-3′-*N*-debenzamidopaclitaxel
Cy5-DBP: cyanine 5 bound to dynein binding peptide
Cy5-KBP: cyanine 5 bound to kinesin binding peptide
*D*eff: effective diffusion constants
FDAP: fluorescence decay after photoactivation
FTX2: flutax-2
GMPCPP: guanosine-5′-[(α,β)-methyleno]­triphosphate
HFX: hexaflutax
HeLaβIII: HeLa cell line overexpressing βIII-tubulin isotype
Kb-3.1: HeLa subclone
Kb-V1: Kb-3.1 overexpressing P-glycoprotein
Kif5B: constitutively active human Kif5B 1-905 kinesin 1
KLC1: kinesin-1 light chain
NMA: normal mode analysis
PAGFP: photoactivatable GFP
PF: protofilament
PgP: P-glycoprotein
PTX: paclitaxel
R/S: resistant to sensitive ratio
TIRF: total internal reflection fluorescence.
