# Supplementary material for: Dissecting Structural and Functional Determinants of Microtubules Stabilization through Guided Chemical Modulation
Source: J Med Chem. 2026 Jul 30;69(15):19317–37. doi: 10.1021/acs.jmedchem.6c01666 (PMC13492380; doi:10.1021/acs.jmedchem.6c01666)
Supplement: Supplementary file 1 [file jm6c01666_si_001.pdf]

Supporting Information

Dissecting structural and functional determinants of microtubules stabilization through guided chemical modulation

Francesca Bonato[1,2§], Rebeca París-Ogáyar[1,3§], Ahmed Soliman[1,4], Óscar Fernández[1], Beatriz Álvarez-Bernad[1], Juan Francisco Giménez-Abián[1], Daniel Lucena-Agell[1], Rafael Hortigüela[1], Lisha Singh[4], Juan Estévez-Gallego[1$], Wei-Shuo Fang[5], Denisa Ondrúšková[6], Valle Palomo[3], Federico Gago[7], Marcus Braun[6], Zdeněk Lánský[6], Shinji Kamimura[8], J. Fernando Díaz[1], Roland Brandt[4], Daniele Passarella[2], María A. Oliva[1*].

[*]To whom correspondence should be addressed: marian@cib.csic.es. [§]These authors contribute equally to this work. [$]Current address: Laboratory of Biomolecular Research, Division of Biology and Chemistry, Paul Scherrer Institut, Villigen (Switzerland)

[1]Unit for the Development of Biological, Immunological and Chemical Drugs. Centro de Investigaciones Biológicas Margarita Salas – CSIC. Madrid. 28040. Spain.

[2]Department of Chemistry, Università degli Studi di Milano. Milan. 20133. Italy.

[3]Unit for the Development of Biological, Immunological and Chemical Drugs. IMDEA Nanociencia. Madrid. 28049. Spain.

[4]Department of Neurobiology, School of Biology/Chemistry, Osnabrück University. Osnabrück. 49076. Germany.

[5]State Key Laboratory of Bioactive Substances and Functions of Natural Medicines & MHC Key Laboratory of Biosynthesis of Natural Products, Institute of Materia Medica, Chinese Academy of Medical Sciences & Pekin Union Medical College. Beijing. 100050. China.

[6]Institute of Biotechnology, Czech Academy of Sciences, BIOCEV. Prague West. 252-50. Czech Republic.

[7]Department of Biomedical Sciences and IQM-UAH Associate Unit. University of Alcalá. Alcalá de Henares. 28805. Spain.

[8]Department of Biological Sciences, Faculty of Science and Engineering, Chuo University. Tokyo. 192-0393. Japan.

Supplementary information

Supplementary Figures S1 – S4.

Supplementary Tables S1 and S2.

Supplementary Movies S1 – S27.

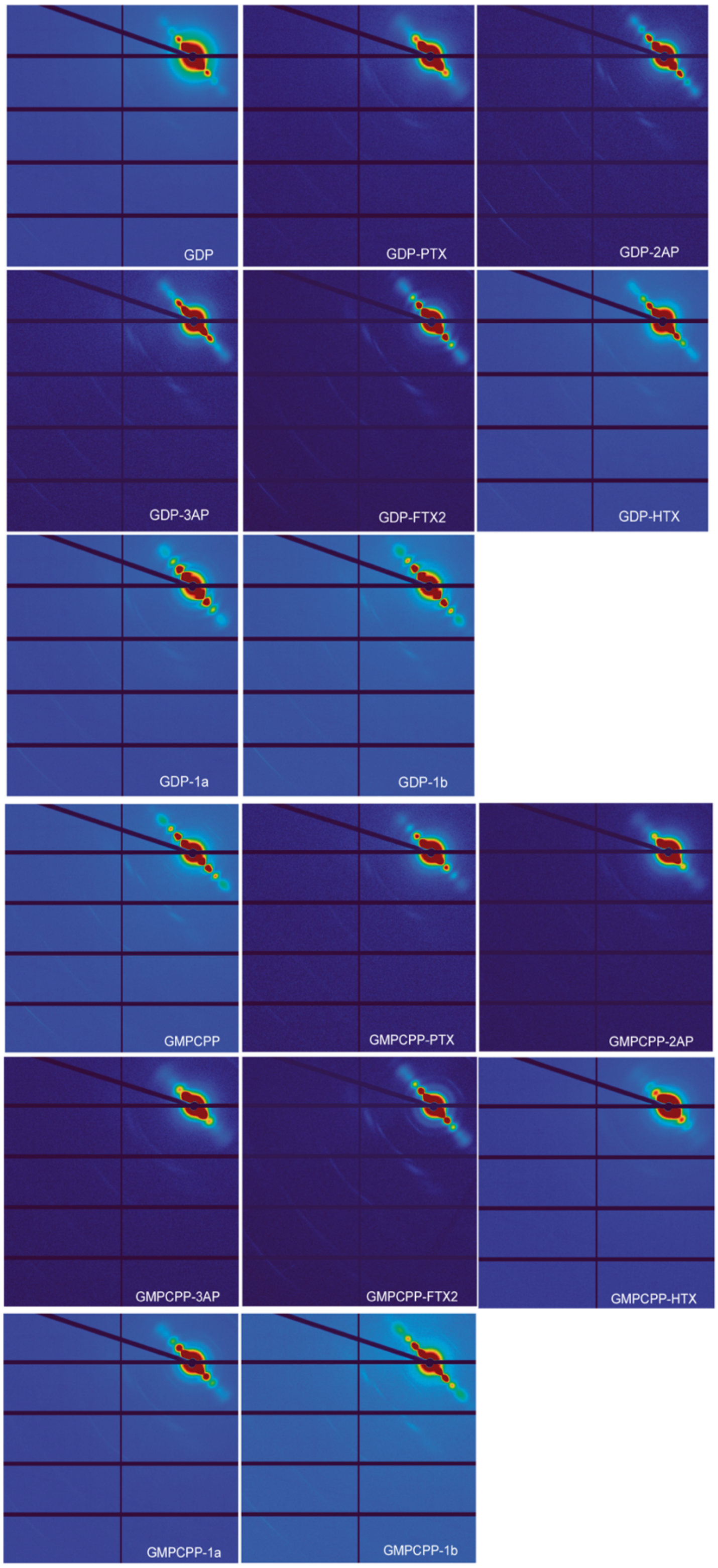

Figure S1: **Shear-flow aligned fiber diffraction images** under all conditions analyzed in this study.

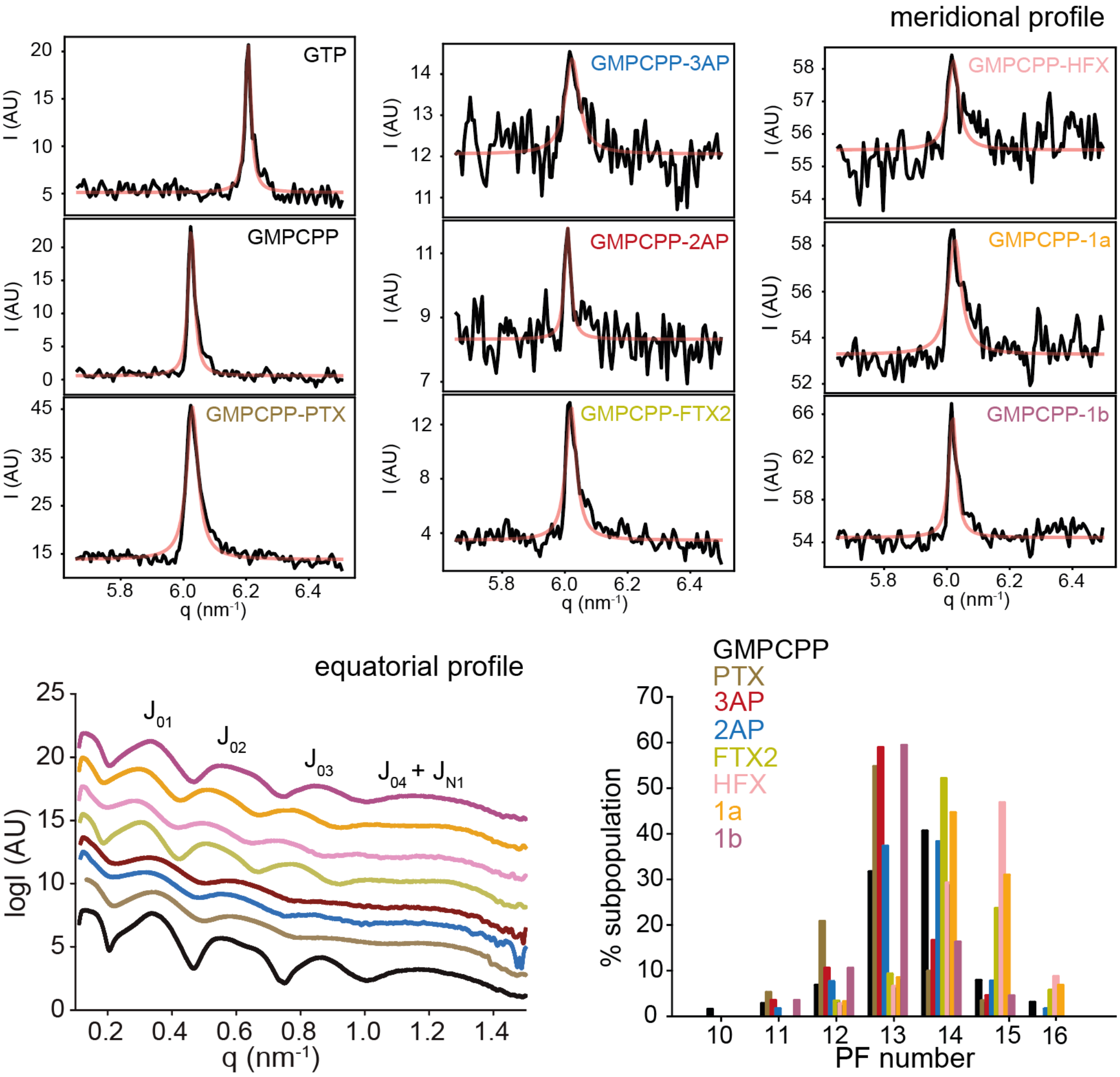

Figure S2: Fiber diffraction profiles of GMPCPP assembled MTs. **Top.** Meridional profiles of MTs assembled in the presence of GMPCPP and stabilized with the different compounds. Profiles are zoom into the 1 nm^{-1} layer band to highlight the differences between expanded and compact lattices (GTP is also represented for comparison). Graphs show raw data (black lines) and analysis fitting (red line). **Below**. Left, equatorial profiles of MTs assembled in the presence of GMPCPP with and without the different compounds. Profiles are shifted in the y axis for clarity and Bessel functions are labeled. Right, distribution of MT subpopulations according to the number of PF in MTs assembled with GMPCPP and MTs further stabilized with the compounds used in this study.

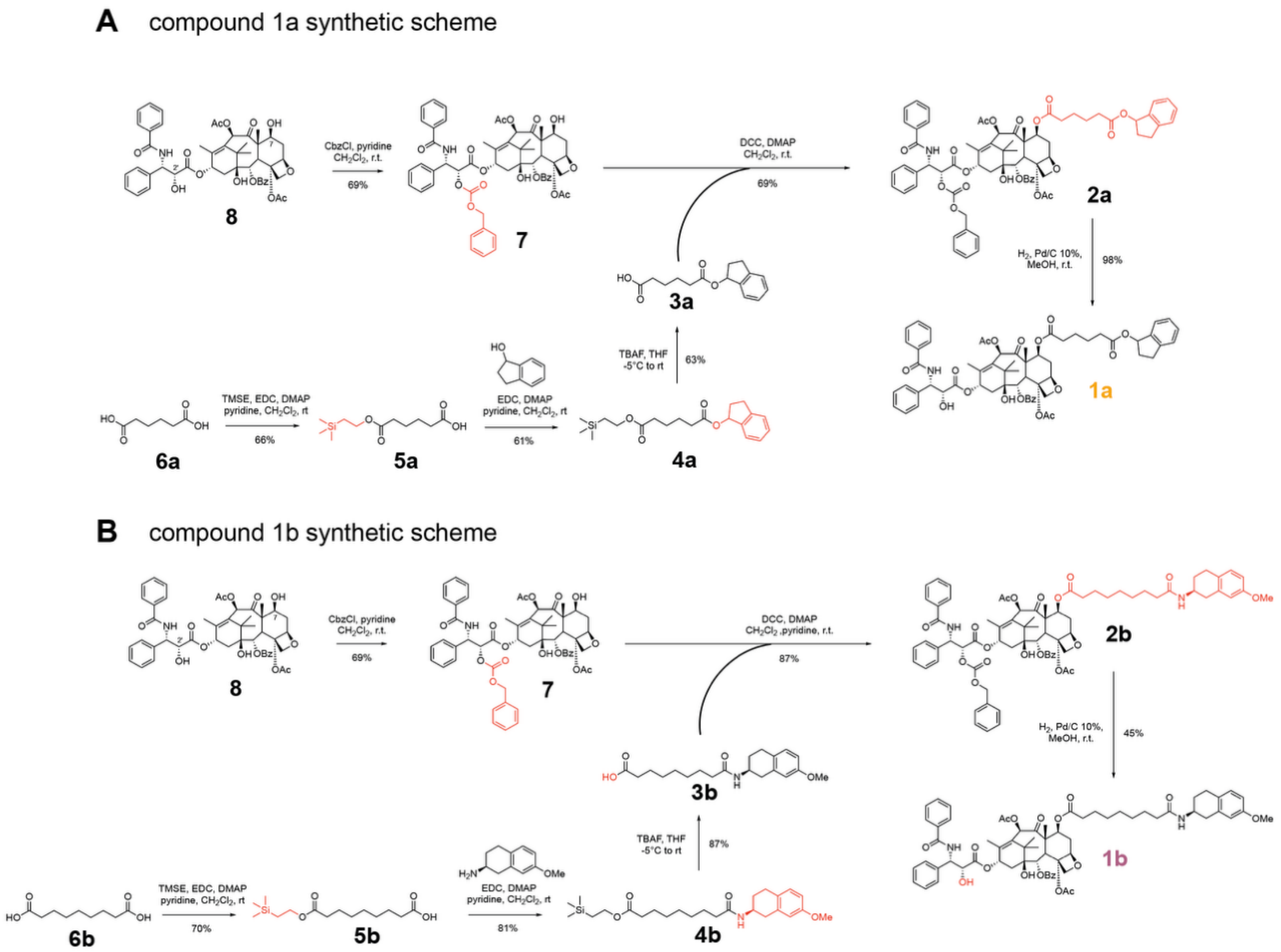

Figure S3: Synthetic scheme of compounds **1a (A)** and compound **1b (B).**

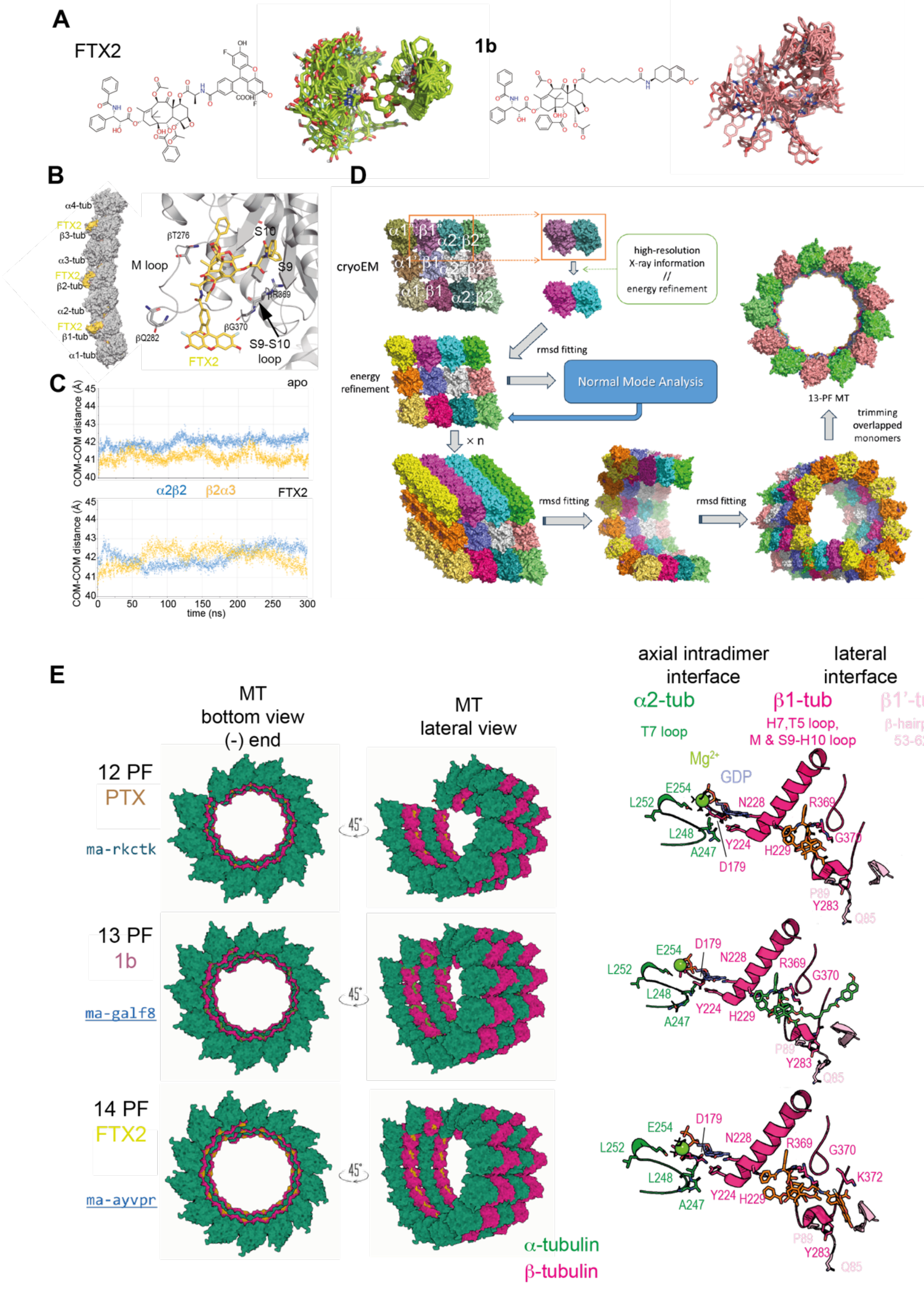

Figure S4: Computational analysis. A. Chemical structure (top) and related MD simulations (bottom) of isolated compounds FTX2 and **1b** in water (water molecules have been removed for clarity). **B.** Left. Surface representation of the PF used in MD simulations and built using three tubulin heterodimers capped by an α-tubulin subunit highlighting FTX2 (yellow) in the taxane site of each β-tubulin. The PF is capped with

an extra α-tubulin subunit to preserve β3-tubulin in a protein-protein axial contact context. Right. Zoom into the taxane site in β2-tubulin (cartoon representation) showing FTX2 (yellow, stick representation) orientation and highlighting main interacting residues from M and S9-S10 loops. **C.** MD of the PF model showing the evolution of the distance between α2-β2 (intradimer, blue) and β2-α3 (interdimer, yellow) in the 300 ns simulation of both, the PF in the absence of ligand at the taxane site (Apo) and, in the presence of FTX2. **D.** Scheme of MT building used in MD and NMA. Briefly, a tubulin dimer from a cryoEM structure (MT patch of 3 PFs with 2 tubulin dimers each, as deposited in the PDB) is optimized considering high-resolution macromolecular crystals information and using computational energy refinement. This refined dimer is placed back into the cryoEM structure using r.m.s.d fitting and the structure is further optimized by energy refinement. Then, the optimized cryoEM patch is reproduced n times and by r.m.s.d fitting the final MT structure is obtained once trimming overlapped monomers. **E.** NMA of PTX bound to 12-PF MT, **1b** bound 13-PF MT and FTX2 bound to 14-PF MT. Left and center panels show bottom (-end) and 45 º rotated lateral views of these MTs in surface representation with α-tubulins in green and β-tubulins in magenta. Right panels display a closer view of β1– α2 axial intradimer interface and β1– β1' lateral interfaces in cartoon representation. The effect of ligand binding to the taxane site translates into changes on longitudinal contacts, following the connected axial network between β1 (taxane site-H7-T5 loop-nucleotide binding site) and α2 T7 loop, and the lateral interactions, between β1 (M loop – S9-S10 loop) and α2 (β–hairpin). Residues contributing to the interfaces are highlighted, together with the bound ligand and de GDP at the E-site (stick representation). NMA reveals that ligand-dependent modulation of the taxane-binding pocket propagates through the M-loop, H7 and T5 loop, and adjacent secondary structural elements, reshaping the lateral and axial network and coupling local ligand recognition to global lattice geometry. Distinct conformational adaptations correlate with the stabilization of specific PF architectures and MT lattice states.

Table S1: Normalized motor probes to control experiments

	Cy5-KBP		Cy5-DBP	
	Distance (nm)	Speed (nms^{-1})	Distance (nm)	Speed (nms^{-1})
**PTX**	0.80 ± 0.02	0.86 ± 0.02	0.81 ± 0.02	0.96 ± 0.03
**FTX2**	0.910 ± 0.003	0.85 ± 0.01	1.06 ± 0.02	1.21 ± 0.02
**1a**	0.92 ± 0.01	0.95 ± 0.01	0.92 ± 0.01	0.98 ± 0.02
**1b**	0.85 ± 0.01	0.87 ± 0.01	0.92 ± 0.01	1.07 ± 0.01

Data are the mean ± SEM values of two independent experiment with two to three different fields analyzed.

Table S2: Fiber diffraction data analysis under assembly TIRF conditions

	Avg. radius[1]	Avg. PF number[2]	1 nm^{-1} band[3]	Avg. monomer rise
PTX_{pre}	11.33±0.07	12.39±0.12	6.02±0.01	4.17±0.01
PTX_{post}	11.52±0.01	12.69±0.01	6.02±0.01	4.17±0.01

data are Mean±SEM

[1]nm as calculated from fitting J_{02}

[2]calculated from interPF mean distance 5.7 nm

[3]2pi nm^{-1} as calculated from 1 nm layer line

Movies

Movie S1: Normal Mode Analysis of a cryoEM patch of 3 PFs with 2 tubulin dimers each.

Movie S2: Movement of Cy5-DBP in SH-SY5Y treated with DMSO

Movie S3: Movement of Cy5-DBP in SH-SY5Y treated with PTX

Movie S4: Movement of Cy5-DBP in SH-SY5Y treated with FTX2

Movie S5: Movement of Cy5-DBP in SH-SY5Y treated with **1a**

Movie S6: Movement of Cy5-DBP in SH-SY5Y treated with **1b**

Movie S7: Movement of Cy5-KBP in SH-SY5Y treated with DMSO

Movie S8: Movement of Cy5-KBP in SH-SY5Y treated with PTX

Movie S9: Movement of Cy5-KBP in SH-SY5Y treated with FTX2

Movie S10: Movement of Cy5-KBP in SH-SY5Y treated with **1a**

Movie S11: Movement of Cy5-KBP in SH-SY5Y treated with **1b**

Movie S12: Movement of Kif5B-EGFP along GMPCPP stabilized MTs.

Movie S13: Movement of Kif5B-EGFP along PTX stabilized MTs.

Movie S14: Movement of Kif5B-EGFP along **1a** stabilized MTs.

Movie S15: Movement of Kif5B-EGFP along **1b** stabilized MTs.

Movie S16: Binding of 10 nM Tau-mCherry along GMPCPP stabilized MTs.

Movie S17: Binding of 40 nM Tau-mCherry along GMPCPP stabilized MTs.

Movie S18: Binding of 10 nM Tau-mCherry along PTXpre stabilized MTs.

Movie S19: Binding of 40 nM Tau-mCherry along PTXpre stabilized MTs.

Movie S20: Binding of 10 nM Tau-mCherry along PTXpost stabilized MTs.

Movie S21: Binding of 40 nM Tau-mCherry along PTXpost stabilized MTs.

Movie S22: Binding of 10 nM Tau-mCherry along FTX2 stabilized MTs.

Movie S23: Binding of 40 nM Tau-mCherry along FTX2 stabilized MTs.

Movie S24: Binding of 10 nM Tau-mCherry along **1a** stabilized MTs.

Movie S25: Binding of 40 nM Tau-mCherry along **1a** stabilized MTs.

Movie S26: Binding of 10 nM Tau-mCherry along **1b** stabilized MTs.

Movie S27: Binding of 40 nM Tau-mCherry along **1b** stabilized MTs.
