# Supplementary material for: Dissecting Structural and Functional Determinants of Microtubules Stabilization through Guided Chemical Modulation
Source: J Med Chem. 2026 Jul 30;69(15):19317–37. doi: 10.1021/acs.jmedchem.6c01666 (PMC13492380; doi:10.1021/acs.jmedchem.6c01666)
Supplement: Supplementary file 2 [file jm6c01666_si_002.pdf]

mple Name: FB44

===

Acq. Operator	: SYSTEM	Seq. Line :	1
Acq. Instrument	: LCMS309	Location :	Vial 1
Injection Date	: 7/2/2026 9:58:35 AM	Inj :	1
		Inj Volume :	10.000 µl

Acq. Method : C:\CHEM32\1\DATA\FRATAX 2026-07-02 09-57-05\FRATAX.M
Last changed : 7/2/2026 9:57:05 AM by SYSTEM
Analysis Method : C:\CHEM32\1\METHODS\NUCLEOTIDESB.M
Last changed : 7/2/2026 1:25:05 PM by SYSTEM

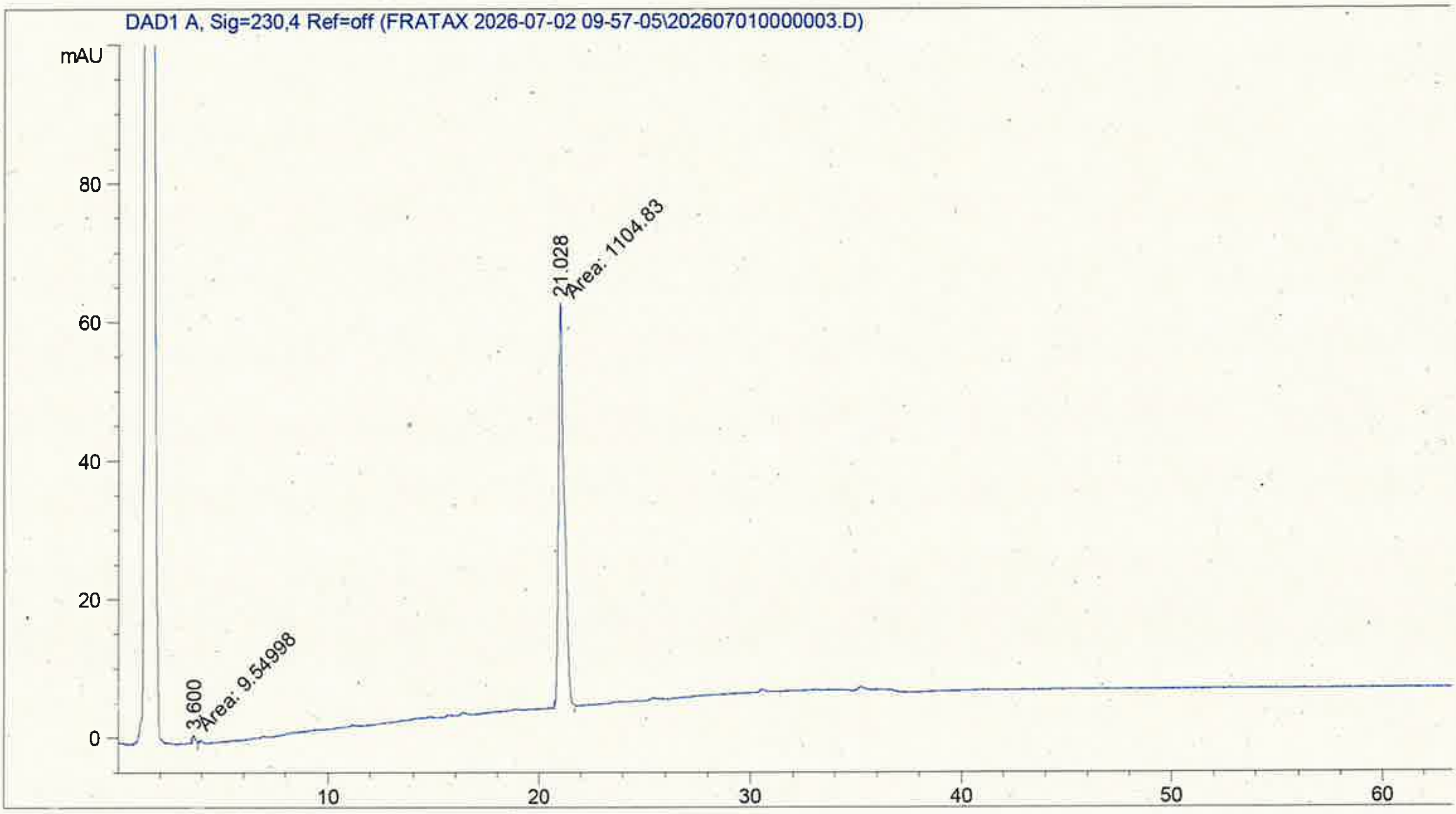

===

Area Percent Report

===

Sorted By : Signal
Multiplier : 1.0000
Dilution : 1.0000
Use Multiplier & Dilution Factor with ISTDs

Signal 1: DAD1 A, Sig=230,4 Ref=off

Peak #	RetTime [min]	Type	Width [min]	Area [mAU*s]	Height [mAU]	Area %
1	3.600	MM	0.1384	9.54998	1.14975	0.8570
2	21.028	MM	0.3156	1104.82910	58.34831	99.1430

Totals : 1114.37909 59.49807

===

*** End of Report ***

===

Acq. Operator	: SYSTEM	Seq. Line :	2
Acq. Instrument	: LCMS309	Location :	Vial 2
Injection Date	: 7/2/2026 11:15:03 AM	Inj :	1
		Inj Volume :	10.000 µl

Acq. Method : C:\CHEM32\1\DATA\FRATAX 2026-07-02 09-57-05\FRATAX.M
Last changed : 7/2/2026 9:57:05 AM by SYSTEM
Analysis Method : C:\CHEM32\1\METHODS\NUCLEOTIDESB.M
Last changed : 7/2/2026 1:22:57 PM by SYSTEM
(modified after loading)

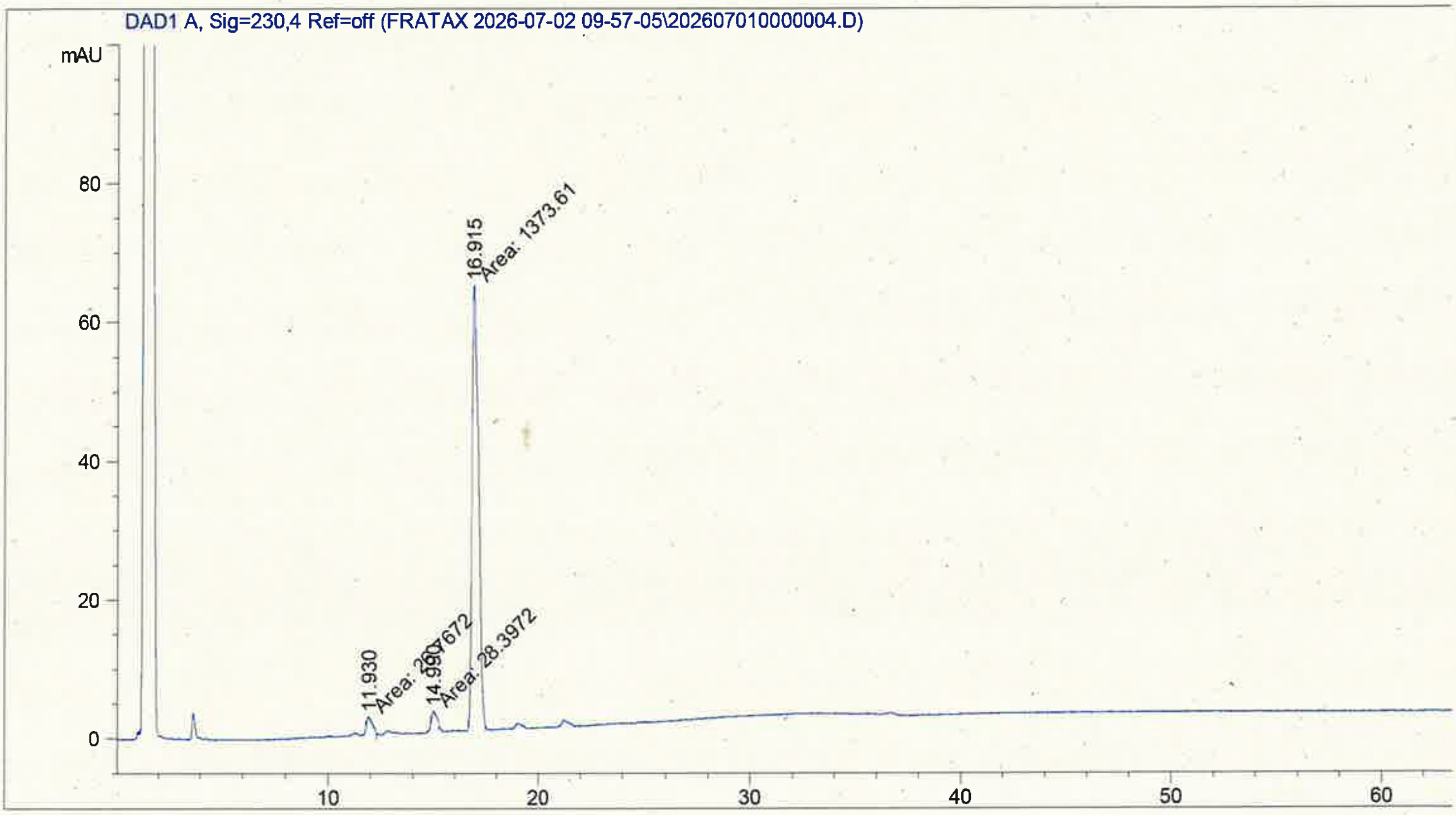

===

Area Percent Report

===

Sorted By : Signal
Multiplier : 1.0000
Dilution : 1.0000
Use Multiplier & Dilution Factor with ISTDs

Signal 1: DAD1 A, Sig=230,4 Ref=off

Peak #	RetTime [min]	Type	Width [min]	Area [mAU*s]	Height [mAU]	Area %
1	11.930	MM	0.2699	26.76722	1.65280	1.8734
2	14.990	MM	0.2430	28.39723	1.94779	1.9875
3	16.915	MM	0.3593	1373.60754	63.72569	96.1390
Totals :				1428.77200	67.32629	
